# Genetic underpinnings of affective temperaments: a pilot GWAS investigation identifies a new genome-wide significant SNP for anxious temperament in *ADGRB3* gene

**DOI:** 10.1038/s41398-021-01436-1

**Published:** 2021-06-01

**Authors:** Xenia Gonda, Nora Eszlari, Dora Torok, Zsofia Gal, Janos Bokor, Andras Millinghoffer, Daniel Baksa, Peter Petschner, Peter Antal, Gerome Breen, Gabriella Juhasz, Gyorgy Bagdy

**Affiliations:** 1 NAP-2-SE New Antidepressant Target Research Group, Hungarian Brain Research Program, Semmelweis University, Budapest, Hungary; 2 Department of Psychiatry and Psychotherapy, Faculty of Medicine, Semmelweis University, Budapest, Hungary; 3 Department of Pharmacodynamics, Faculty of Pharmacy, Semmelweis University, Budapest, Hungary; 4 Department of Forensic and Insurance Medicine, Faculty of Medicine, Semmelweis University, Budapest, Hungary; 5 Department of Measurement and Information Systems, Budapest University of Technology and Economics, Budapest, Hungary; 6 Abiomics Europe Ltd, Budapest, Hungary; 7 SE-NAP 2 Genetic Brain Imaging Migraine Research Group, Hungarian Brain Research Program, Semmelweis University, Budapest, Hungary; 8 Bioinformatics Center, Institute for Chemical Research, Kyoto University, Uji, Japan; 9 Social, Genetic and Developmental Psychiatry Centre, King’s College London, London, UK

**Keywords:** Depression, Human behaviour

## Abstract

Although recently a large-sample GWASs identified significant loci in the background of depression, the heterogeneity of the depressive phenotype and the lack of accurate phenotyping hinders applicability of findings. We carried out a pilot GWAS with in-depth phenotyping of affective temperaments, considered as subclinical manifestations and high-risk states for affective disorders, in a general population sample of European origin. Affective temperaments were measured by TEMPS-A. SNP-level association was assessed by linear regression models, assuming an additive genetic effect, using PLINK1.9. Gender, age, the first ten principal components (PCs) and the other four temperaments were included in the regression models as covariates. SNP-level relevances (*p*-values) were aggregated to gene level using the PEGASUS method^1^. In SNP-based tests, a Bonferroni-corrected significance threshold of *p* ≤ 5.0 × 10^−8^ and a suggestive significance threshold of *p* ≤ 1.0 × 10^−5^, whereas in gene-based tests a Bonferroni-corrected significance of 2.0 × 10^−6^ and a suggestive significance of *p* ≤ 4.0 × 10^−4^ was established. To explore known functional effects of the most significant SNPs, FUMA v1.3.5 was used. We identified 1 significant and 21 suggestively significant SNPs in *ADGRB3*, expressed in the brain, for anxious temperament. Several other brain-relevant SNPs and genes emerged at suggestive significance for the other temperaments. Functional analyses reflecting effect on gene expression and participation in chromatin interactions also pointed to several genes expressed in the brain with potentially relevant phenotypes regulated by our top SNPs. Our findings need to be tested in larger GWA studies and candidate gene analyses in well-phenotyped samples in relation to affective disorders and related phenotypes.

## Introduction

Depression is a severe illness causing significant dysfunction and suffering. Unlike other illnesses where there is significant new progress and paradigm shifts in therapy also due to understanding and exploiting genetic variation associated with the illness, there is, with the exception of the promising introduction of glutamatergic approaches, a void of new approaches to understanding and treatment of affective disorders contributing to depression predicted to be the illness associated with the highest disease burden in the next very few years^2^.

One reason for our lack of sufficient insight into the etiopathology and neurobiology of depression is the failure to identify replicable genetic variation associated with the emergence and clinical characteristics of this illness. Although candidate gene studies targeted a large number of possible variants, only a few of these were investigated in at least three studies and even less were replicated^3^. Genome-wide analysis approaches similarly yielded less results in case of depression compared to other psychiatric illnesses and, where suggestive significant results were retrieved, these did not confirm the role of previous candidate genes, nor could they be replicated in subsequent genome-wide association studies (GWASs). However, the most recent large-sample GWAS attempts involving joint databases and megasamples at the sacrifice of accurate phenotyping managed to identify a few significant variants with 102 significant hits in the latest study^4^.

However, the lack of in-depth and precise phenotyping may be a crucial weakness of such studies, as depression as a disease category is a highly heterogeneous phenomenon, where divergent clinical manifestations may equally be labelled as depressive disorder without a single overlapping symptom^5^, and in the background of such heterogeneous clinical symptoms divergent neurobiological pathways and distinct genetic variation are likely to play a role^6^. Subtypes of depression with clinically highly distinct features, such as unipolar or bipolar depression^7^, alexythmic depression^8^, melancholic and atypical depression^9,10^, or depression associated with increased suicidal risk^10^ to name only a few, require focusing on different psychosocial or neurobiological targets, and for this, understanding their distinctive genetic determinants would provide the missing background and first step. Given the lack of proper efficacy of current, monoamine-based treatment approaches to depression, not only new pharmacological targets would be needed but novel agents need to be matched to more clear-cut illness phenotypes within the heterogeneous depression group^6^.

One possible approach to understanding depression via a more homogeneous categorization is the use of endophenotypes related to mood disorders, which are, by definition, better characterized and can be more closely mapped to singular neurobiological alterations^11^. Affective temperaments, thought to possess a strong biological background, manifesting in an early age and persisting through the life span, and being closely linked to affective disorders constituting in their more dominant manifestation a high-risk state or the subclinical form of affective disorders, and possessing a strong pathoplastic role, offer themselves as possible endophenotypes^12,13^. Previous research indicated their genetic associations, strong heritability, more marked manifestation not only in affective disorder patients but also in their first-degree relatives, dimensional distribution in the population, and a strong association with several outcomes of affective illness and general illness course^13^, which argue for using them in GWASs to bring us closer to the identification of genes in depression with an in-depth and precise endophenotyping. Yet, previously, no whole-genome-wide analysis was performed for affective temperaments in a general population sample and there has been only one GWAS in a bipolar patient sample^14^.

The aim of our present study was to perform a pilot GWAS on affective temperaments in a European general population sample.

## Methods

### Participants

The study was part of the NewMood project (New Molecules in Mood Disorders, Sixth Framework Program of the EU, LSHM-CT-2004-503474) funded by the European Union. NewMood has been a collaboration between 13 clinical and basic science research groups, across 10 European countries aimed at identifying new molecules in the background of mood disorders (www.newmood.co.uk), focusing on genetics with the specific aim of demonstrating allelic variation in association with vulnerability markers of depression, translation from rodent to human models, and specifically using translational endophenotypic markers rather than symptoms or diagnoses^15–17^. In the human clinical part of the project over 3000 participants of European White ethnic origin aged 18–60 years, in Greater Manchester and Budapest, were recruited via advertisements, general practices and a website. Participants filled out a questionnaire pack for precise and in-depth phenotyping of multiple endophenotypes related to susceptibility to depression, provided detailed background information on personal and family psychiatric history, as well as demographic and socioeconomic circumstances, and provided DNA using a saliva sampling kit. In the present study, we used phenotypic and genotypic data from 775 participants in the Budapest cohort. All procedures were carried out in accordance with the Declaration of Helsinki and were approved by the Scientific and Research Ethics Committee of the Medical Research Council, Budapest, Hungary. All participants provided a written informed consent prior to participating in the study.

### Phenotype

Participants filled out the NewMood questionnaire pack, including the standardized Hungarian version of the 110-item Temperament Evaluation of Memphis, Pisa, Paris and San Diego (TEMPS-A) questionnaire to measure affective temperaments^12,18^ and a background questionnaire including questions on age, gender, previous psychiatric illness, family history of psychiatric illness, somatic disorders and relevant demographic information. The TEMPS-A consists of five scales measuring the five affective temperaments described by Akiskal based on a clinical population and their first-degree relatives, including the depressive, cyclothymic, hyperthymic, irritable and anxious temperaments. Each affective temperament score was calculated as a continuous weighted score by dividing the sum of item scores by the number of completed items.

### Genotyping, quality control and imputation

Participants provided DNA by a genetic saliva sampling kit. Genomic DNA was extracted from buccal mucosa cells according to established protocols^19^. Genotyping was performed using Illumina’s CoreExom PsychChip, yielding a total of 573,141 variants, the genomic positions of which were defined according to the build GRCh37/hg19. Quality control and imputation was based on refs. ^20,21^ (see Supplementary File 1).

### Statistical analyses

Descriptive statistics were done with SPSS25.

Primary single-nucleotide polymorphism (SNP)-based association tests for each affective temperament phenotype were calculated using linear regression models in Plink 1.9 (https://www.cog-genomics.org/plink2), assuming an additive genetic effect. All models contained the first ten calculated principal components (PCs), gender, age, and the other four temperaments as covariates.

Gene-based tests for each affective temperament phenotype were calculated using Pegasus^1^. Significance (*p*-value) of genes is calculated by a method that aggregates variant-level *p*-values and takes into account the dependence between them (induced by the linkage between the respective variants) by applying a null model of a multivariate normal distribution with a covariance matrix reflecting the aforementioned linkage.

To explore the known functional effects of our most significant SNPs as reported in public open databases based on expression quantitative trait loci (eQTL) and three-dimensional chromatin interaction, we used FUMA v1.3.5^22^, with a *p* ≤ 1 × 10^−5^ threshold for lead SNPs, an *R*^2^ ≥ 0.5 to define a genomic risk locus around a lead SNP, and a *p* ≤ 0.05 to involve SNPs into it. Each SNP of the genomic risk loci (referred to as top SNPs or our most significant SNPs) were mapped to a gene if either residing within gene boundaries extended by 10,000 base pairs, or having a false discovery rate *q* ≤ 0.05 with it in the external eQTL, or a *q* ≤ 1 × 10^−6^ with its promoter region in the external chromatin interaction dataset^22^.

## Results

### Characteristics of the sample

Imputation and quality-control steps yielded 2,550,710 SNPs and 775 subjects with data on the 5 affective temperaments, gender, age, and quality-controlled genomic data. By convention the threshold for statistical significance is based on a Bonferroni correction for 1 million comparisons, yielding a threshold of *p* ≤ 5.0 × 10^−8^, whereas at the SNP-level *p* ≤ 1.0 × 10^−5^ was determined as threshold for suggestive significance^23^. For gene-based tests, a Bonferroni-corrected significance threshold of 2.0 × 10^−6^ was applied and, at the gene level, *p* ≤ 4.0 × 10^−4^ was determined as threshold for suggestive significance, the former one corresponding to the Bonferroni-corrected nominal significance level of 0.05 assuming 25,000 independent tests (the approximate number of the genes tested) and the latter one corresponding to that number multiplied by 200 (as in the case of variant-level tests).

Descriptive statistics on affective temperament scores, gender, age, self-reported psychiatric history and treatment are provided in Supplementary Table S1. Affective temperament scales were significantly and at least moderately correlated with each other in our sample (Supplementary Table S2); thus, when analysing specific variability of each affective temperament scale, the other four temperament scales were covariates in the model.

### SNPs in the background of the five affective temperaments

#### Significant and suggestively significant findings in SNP-based tests for anxious temperament

**Fig. 1:**
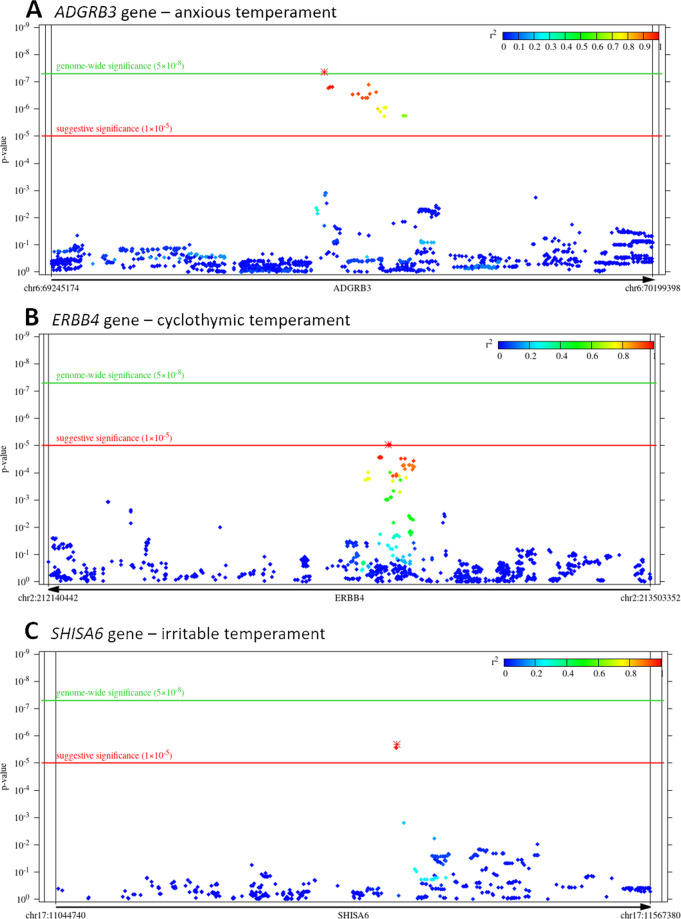
Zoomed Manhattan plots of significant or suggestively significant SNPs for anxious, cyclothymic and irritable temperaments. Zoomed Manhattan plots of **A** *ADGRB3* gene on chromosome 6 for anxious temperament as the outcome phenotype (*rs3798978* survived correction for genome-wide significance (*p* = 4.44 × 10^−8^), whereas 21 other SNPs showed suggestive significance); **B** *ERBB4* gene on chromosome 2 for cyclothymic temperament as the outcome phenotype (4 SNPs showed suggestive significance); and **C** *SHISA6* gene on chromosome 17 for irritable temperament as the outcome phenotype (2 SNPs showed suggestive significance). *P*-value is displayed in function of genomic position for each single-nucleotide polymorphism (SNP) in the region. Colours denote the *r*^2^-value of linkage disequilibrium (LD) with the most significant SNP (marked with asterisk). Gene boundaries and their extension by 10,000 base pairs (as defined for the gene-based tests) are marked with vertical lines.

**Fig. 2:**
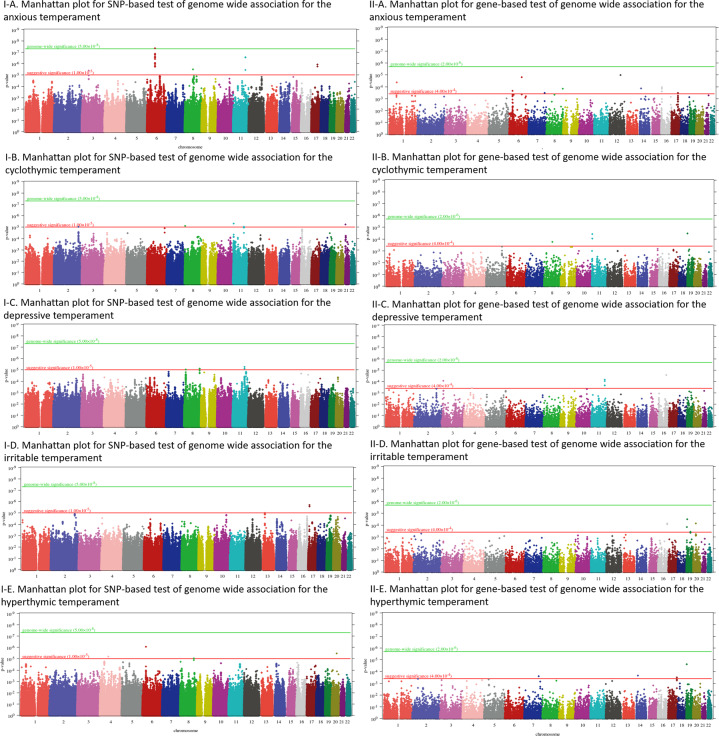
Manhattan plots of genome-wide SNP-based and gene-based tests for the five affective temperaments. Manhattan plots of genome-wide SNP-based tests (**I**) and gene-based tests (**II**) for anxious (**I-A**, **II-A**), depressive (**I-B**, **II-B**), cyclothymic (**I-C**, **II-C**), irritable (**I-D**, **II-D**) and hyperthymic (**I-E**, **II-E**) temperaments as outcome. *P*-value is displayed in function of genomic position for each single-nucleotide polymorphism (SNP). The red and green lines denote the levels of a suggestive and a genome-wide significance, respectively. *P*-value is displayed in function of genomic position for each single-nucleotide polymorphism (SNP). The red and green lines denote the levels of a suggestive and a genome-wide significance, respectively.

With respect to specific SNPs, SNP-based association tests yielded a genomic inflation estimate (based on median *χ*^2^) of *λ* = 1.00703 for anxious temperament. For the quantile–quantile (QQ) plot, see Supplementary Fig. S1. In case of anxious temperament, rs3798978 within the *ADGRB3* gene survived correction for genome-wide significance (*p* = 4.44 × 10^−8^), on chromosome 6, whereas 21 other SNPs within *ADGRB3* (Fig. 1A) and 7 other SNPs in intergenic regions on chromosome 8, 11, and 17 had a suggestive significance after correction (Fig. 2 I-A and Table 1).

**Table 1:** Most significant SNPs with genome-wide or suggestive significances for the five affective temperaments.

Chromosome	SNP	Base position	Minor/effect allele	*β*	*P*	Genes
Anxious temperament						
3	rs1281465	59,664,687	G	0.03152	4.03E − 06	*LOC339902*
** 6**	**rs3798978**	**69,678,213**	**G**	**0.08709**	**4.44E** − **08**	***ADGRB3***
6	rs34526480	69,684,542	G	0.08332	1.73E − 07	*ADGRB3*
6	rs13191706	69,686,817	G	0.08362	1.58E − 07	*ADGRB3*
6	rs13194716	69,686,889	G	0.08362	1.58E − 07	*ADGRB3*
6	rs71555397	69,690,915	G	0.08362	1.58E − 07	*ADGRB3*
6	rs3799007	69,723,045	C	0.08099	2.85E − 07	*ADGRB3*
6	rs117667441	69,731,898	T	0.08154	2.76E − 07	*ADGRB3*
6	rs80164607	69,737,887	T	0.07958	3.95E − 07	*ADGRB3*
6	rs79911876	69,743,496	T	0.07958	3.95E − 07	*ADGRB3*
6	rs75556966	69,746,343	T	0.07958	3.95E − 07	*ADGRB3*
6	rs74802157	69,748,723	T	0.08445	1.30E − 07	*ADGRB3*
6	rs118099456	69,750,540	G	0.08279	2.71E − 07	*ADGRB3*
6	rs3799021	69,760,328	G	0.08314	2.41E − 07	*ADGRB3*
6	rs62416781	69,762,935	A	0.07648	9.92E − 07	*ADGRB3*
6	rs62416782	69,766,653	A	0.07518	1.28E − 06	*ADGRB3*
6	rs62416803	69,766,783	T	0.07518	1.28E − 06	*ADGRB3*
6	rs3734465	69,772,650	G	0.07542	8.95E − 07	*ADGRB3*
6	rs62416807	69,773,007	G	0.07437	1.88E − 06	*ADGRB3*
6	rs3799023	69,774,810	C	0.07542	8.95E − 07	*ADGRB3*
6	rs79811527	69,776,038	T	0.07542	8.95E − 07	*ADGRB3*
6	rs3799030	69,803,044	A	0.07696	1.81E − 06	*ADGRB3*
6	rs62406773	69,806,494	T	0.0774	1.75E − 06	*ADGRB3*
8	rs13251367	72,001,931	A	0.03391	3.26E − 06	*XKR9* | | *EYA1*
11	rs484334	107,769,407	C	−0.03474	2.90E − 07	*SLC35F2* | | *LOC643949*
11	rs563811	107,772,104	A	−0.0347	2.72E − 07	*SLC35F2* | | *LOC643949*
11	rs552905	107,777,210	A	−0.03163	3.55E − 06	*SLC35F2* | | *LOC643949*
17	rs7226241	53,573,452	A	0.05233	1.20E − 06	MMD | | *TMEM100*
17	rs1553677	53,573,902	A	0.05127	1.77E − 06	*MMD* | | *TMEM100*
Cyclothymic temperament						
2	rs73988952	212,909,118	A	−0.05511	9.17E − 06	*ERBB4*
2	rs73988954	212,911,142	C	−0.05511	9.17E − 06	*ERBB4*
2	rs141189957	212,912,423	C	−0.05511	9.17E − 06	*ERBB4*
2	rs10445735	212,913,183	G	−0.05511	9.17E − 06	*ERBB4*
8	rs2656285	4,117,538	A	−0.03344	7.80E − 06	*CSMD1*
11	rs7948848:	2,981,896	A	−0.03195	4.72E − 06	*NAP1L4. SNORA54*
11	rs11020416	93,293,727	C	0.03278	9.79E − 06	*LOC729466* | | *LOC642897*
11	rs2895473	93,293,780	A	0.03273	9.81E − 06	*LOC729466* | | *LOC642897*
11	rs4753475	93,295,861	A	0.03274	9.86E − 06	*LOC729466* | | *LOC642897*
11	rs60944979	93,297,230	C	0.03295	8.93E − 06	*LOC729466* | | *LOC642897*
11	rs4753086:93297625	93,297,625	C	0.03284	9.61E − 06	*LOC729466* | | *LOC642897*
21	rs2823289	16,841,195	G	0.03159	5.70E − 06	*NRIP1* | | *CYCSP42*
Depressive temperament						
8	rs34835594	14,570,438	A	0.02743	9.14E − 06	*SGCZ*
8	rs7831625	135,076,495	T	0.04273	7.56E − 06	*LOC100129104* | | *ZFAT*
8	rs7836140	135,077,157	A	0.04273	7.56E − 06	*LOC100129104* | | *ZFAT*
8	rs16905065	135,077,484	G	0.04273	7.56E − 06	*LOC100129104* | | *ZFAT*
11	rs1917447	103,498,125	C	−0.02727	7.56E − 06	*DYNC2H1* | | *PDGFD*
11	rs1917448	103,498,150	G	−0.02761	5.26E − 06	*DYNC2H1* | | *PDGFD*
Irritable temperament						
13	rs9542430	35,400,570	T	0.03006	9.73E − 06	*LOC100129452* | | *NBEA*
17	rs3111836	11,344,043	G	0.03776	2.74E − 06	*SHISA6*
17	rs2969184	11,344,615	A	0.03824	2.02E − 06	*SHISA6*
Hyperthymic temperament						
4	rs2123430	71,999,712	A	0.04798	6.51E − 06	*LOC100128311* | | *LOC727995*
4	rs11729471	72,000,800	A	0.04829	6.40E − 06	*LOC100128311* | | *LOC727995*
6	rs3131004	31,095,294	G	−0.04087	9.00E − 07	*CDSN.PSORS1C1*
8	rs1793704	118,307,005	T	0.03791	9.23E − 06	*SLC30A8* | | *MED30*
20	rs75945142	41,850,477	A	−0.0455	3.31E − 06	

*p*-value; Bonferroni-corrected *p*-level for significance: *p* ≤ 5 × 10^−8^. *P*-level of suggestive significance: *p* ≤ 1 × 10^−5^. Significant hits are marked with bold.

#### Suggestively significant findings in SNP-based tests for cyclothymic temperament

For cyclothymic temperament, *λ*-value resulting from genome-wide SNP-based tests was *λ* = 1.00000. For the QQ plot, see Supplementary Fig. S2. No SNP survived Bonferroni correction for multiple testing; however, 12 SNPs showed a suggestive significance, 4 of which reside in *ERBB4* (Fig. 1B), whereas the others reside in genes *CSMD1*, *NAP1L4*, *SNORA54* or are intergenic (Fig. 2 I-B and Table 1).

#### Suggestively significant findings in SNP-based tests for depressive temperament

In case of depressive temperament, genome-wide SNP-based tests yielded a genomic inflation factor of *λ* = 1.00172. For the QQ plot, see Supplementary Fig. S3. No SNP survived Bonferroni correction for multiple testing, but five SNPs showed a suggestive significance, one of which resides in the *SGCZ* gene, whereas the rest are intergenic (Fig. 2 I-C and Table 1).

#### Suggestively significant findings in SNP-based tests for irritable temperament

In case of irritable temperament, genome-wide SNP-based tests yielded a genomic inflation factor of *λ* = 1.00000. For the QQ plot, see Supplementary Fig. S4. No SNP survived correction for multiple testing; however, three SNPs showed a suggestive significance (Fig. 2 I-D and Table 1). Except for one intergenic SNP, the other two SNPs are mapped to the *SHISA6* gene (Fig. 1C).

#### Suggestively significant findings in SNP-based tests for hyperthymic temperament

In case of hyperthymic temperament, genome-wide SNP-based tests yielded a genomic inflation factor of *λ* = 1.02018. For the QQ plot, see Supplementary Fig. S5. No SNP survived Bonferroni correction for multiple testing, but five SNPs showed a suggestive significance that either reside in genes *CDSN*, *PSORS1C1*, or are intergenic (Fig. 2 I-E and Table 1).

### Suggestively significant findings in gene-based tests for affective temperament phenotypes

Hits with genome-wide suggestive *p*-values at the gene level for each temperament together with the most significant SNP within each gene are shown in Table 2 and Fig. 2-II. No genes reached genome-wide significance after correction for multiple testing in case of any of the temperaments; however, several genes with suggestive significance were identified for each affective temperament.

**Table 2:** Genes with genome-wide suggestive significance and most significant SNPs for the five affective temperaments.

Gene	*p*	No. of SNPs	Best SNP	SNP *p*-value
Anxious temperament				
* INSL5*	4.23E − 05	13	rs1353716	3.05E − 05
* FYN*	1.60E − 05	225	rs1409839	1.22E − 05
* HCG22*	0.00021981	194	rs2905757	7.83E − 05
* PIP*	0.00032626	27	rs10270005	8.70E − 05
* MIR1251*	1.02E − 05	7	rs4418855	0.0001149
* HECTD1*	0.000138739	49	rs11625570	5.74E − 05
* ROGDI*	0.000245947	5	rs9673735	0.0001447
* SEPT12*	0.000105394	6	rs4389143	5.22E − 05
* SMIM22*	0.00014637	8	rs4389143	5.22E − 05
* MIR454*	0.000333644	10	rs8077052	0.0003431
Cyclothymic temperament				
* C8orf89*	0.000172857	8	rs2925445	0.0001995
* NAP1L4*	9.29E − 05	107	rs7948848	4.72E − 06
* SNORA54*	3.75E − 05	46	rs7948848	4.72E − 06
Depressive temperament				
* MIR4270*	8.68E − 05	30	rs9821793	3.73E − 05
* UGT2B7*	0.000363155	345	rs4694604	0.0002398
* LOC100652768*	9.10E − 05	39	rs634960	1.43E − 05
* PCSK7*	0.000214682	53	rs634960	1.43E − 05
* SIDT2*	0.000214728	46	rs634960	1.43E − 05
* TAGLN*	6.61E − 05	42	rs634960	1.43E − 05
Irritable temperament				
* CENPN*	7.23E − 05	50	rs2602428	1.97E − 05
* CMC2*	8.50E − 05	71	rs7201499	2.76E − 05
* MBD3L3*	3.05E − 05	2	rs2967642	0.000118
* ZNF566*	0.000364777	125	rs2385795	1.75E − 05
* ZNF77*	0.000148404	2	rs11666431	0.0001534
* ENTPD6*	7.07E − 05	54	rs6115093	4.27E − 05
Hyperthymic temperament				
* LOC100506682*	0.000247609	65	rs10278458	0.0002187
* SNX6*	0.00022356	55	rs28421851	2.67E − 05
* CANT1*	0.000306978	14	rs12450407	3.98E − 05
* ZNF77*	2.31E − 05	2	rs11666431	0.0001303

*p*-value: empirical *p*-value based on one million tests; Bonferroni-corrected *p*-level for significance *p* ≤ 2 × 10^−6^. Suggestive level of significance *p* ≤ 4 × 10^−4^.

#### Suggestively significant findings in gene-based tests for anxious temperament

In case of anxious temperament, for the QQ plot, see Supplementary Fig. S6; *INSL5*, *FYN, HCG22*, *PIP*, *MIR1251*, *HECTD1*, *ROGDI*, *SEPT12*, *SMIM22* and *MIR454* genes survived the threshold for suggestive significance after correction for multiple testing (Table 2 and Fig. 2 II-A).

#### Suggestively significant findings in gene-based tests for cyclothymic temperament

In case of cyclothymic temperament, for the QQ plot, see Supplementary Fig. S7; *C8orf89*, *NAP1L4* and *SNORA54* genes survived the threshold for suggestive significance after correction for multiple testing (Table 2 and Fig. 2 II-B).

#### Suggestively significant findings in gene-based tests for depressive temperament

In case of depressive temperament, for the QQ plot, see Supplementary Fig. S8; after correction for multiple testing, *MIR4270*, *UGT2B7*, *LOC100652768*, *PCSK7*, *SIDT2* and *TAGLN* genes remained suggestively significant (Table 2 and Fig. 2 II-C).

#### Suggestively significant findings in gene-based tests for irritable temperament

In case of irritable temperament, for the QQ plot, see Supplementary Fig. S9; *CENPN*, *CMC2*, *MBD3L3*, *ZNF566*, *ZNF77* and *ENTPD6* genes remained suggestively significant after correction for multiple testing (Table 2 and Fig. 2 II-D).

#### Suggestively significant findings in gene-based tests for hyperthymic temperament

In case of hyperthymic temperament, for the QQ plot, see Supplementary Fig. S10; *LOC100506682*, *SNX6*, *CANT1* and *ZNF77* were suggestively significant after correction for multiple testing (Table 2 and Fig. 2 II-E).

### Genomic location of top SNPs for affective temperament phenotypes

**Fig. 3:**
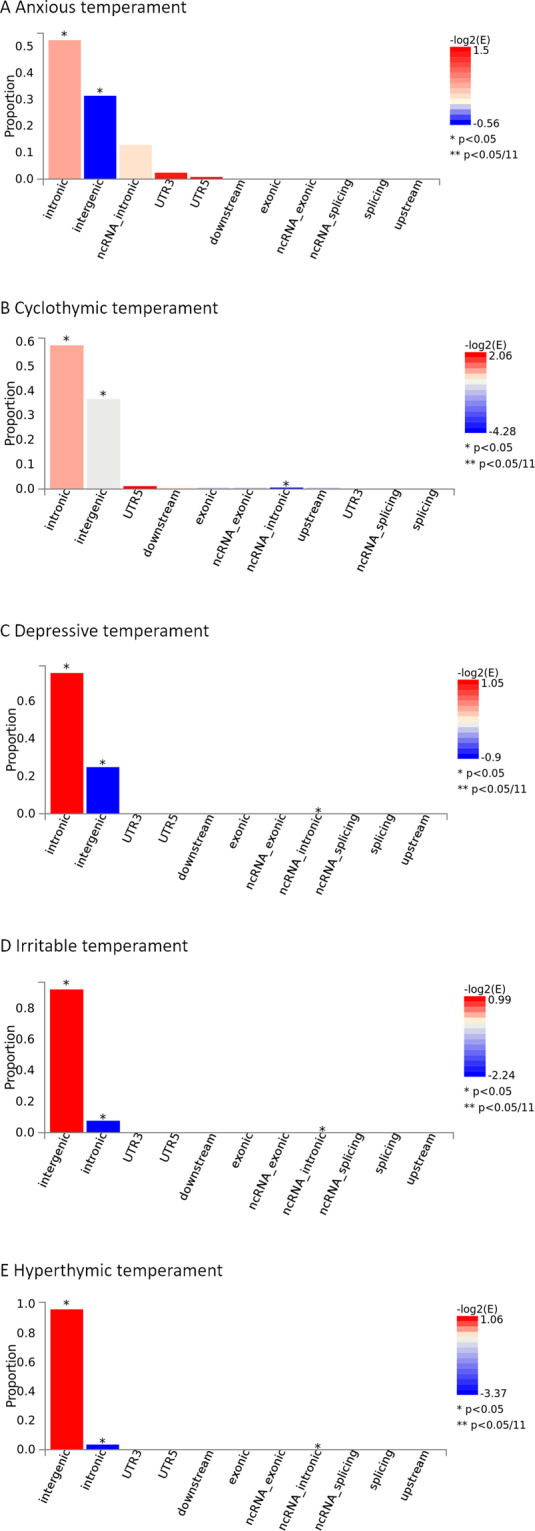
Genomic location and functional consequences of SNPs for affective temperament phenotypes. Histogram of functional consequences of SNPs for anxious (**A**), cyclothymic (**B**), depressive (**C**), irritable (**D**), and hyperthymic (**E**) temperaments.

Proportion of intronic SNPs was nominally significant in case of all five affective temperament phenotypes, and with the exception of anxious temperament phenotype, in case of the other four temperament phenotypes proportion of noncoding RNA intronic SNPs was also nominally significant (Fig. 3). In case of all affective temperament phenotypes, proportion of intronic SNPs, and, with the exception of anxious temperament, proportion of noncoding RNA intronic SNPs were significant, thus, the majority of identified SNPs likely influence function of protein- or RNA-coding genes by mechanisms other than amino acid replacement.

### Functional effects of the top SNPs identified for affective temperament phenotypes on gene expression regulation in the brain

We carried out functional mapping and annotation concerning genes regulated in all tissues and, as we investigated a psychological phenotype, specifically in the brain, by our top SNPs, based on external chromatin interaction databases and eQTL databases GTEx v6 and v7^24,25^, and BRAINEAC^26^, which comprise several brain regions, as well as xQTLServer^27^ and CommonMind Consortium (CMC)^28^ samples, which encompass only the dorsolateral prefrontal cortex (DLPFC). FUMA results in case of the five investigated affective temperament phenotypes concerning regulated genes in all tissues and cell types available are shown in Supplementary Tables S3–S12 and Supplementary Figs. S11–S23. Here we only describe and discuss functional effects of top SNPs in case of each affective temperament phenotypes in the brain; however, effects on all tissue types are shown in Supplementary Tables and Figs.

#### Functional effects of top SNPs identified for the anxious temperament phenotype

We found that top SNPs for the anxious temperament phenotype on chromosome 11 were associated with gene expression levels in *SLC35F2* in the DLPFC (in CMC samples), in the hypothalamus (in GTEx/v7 samples), in nucleus accumbens basal ganglia (in GTEx/v7 samples) and in the pituitary (in GTEx/v7 samples); in *ACAT1* (in GTEx/v6 samples) and *SLN* (GTEx/v7 samples) in the cerebellum; and in *ELMOD1* in the DLPFC (in CMC samples) (Supplementary Table S1 and Supplementary Fig. S13). Top SNPs for the anxious temperament took part in chromatin interactions in the fetal cortex with *Lnc-NPAT-2*:*ACAT*; *NPAT*:*ATM*:*Y_RNA*; *SLN*:*ENSG00000268602*; *ENSG00000214306:ENSG00000214305* (Metazoa_SRP) in Giusti-Rodriguez et al.^29^; and in neural progenitor cells with *RPL37P15*, *NPM1P37* and *MMD* in GS87112 samples (Supplementary Table S4).

#### Functional effects of top SNPs identified for the cyclothymic temperament phenotype

FUMA has indicated that top SNPs in case of cyclothymic temperament affected expression levels on chromosome 11 in *AC131971.1* in the cortex (GTEx/V7 samples) and expression levels in *C11orf54* in the thalamus (BRAINEAC samples), and were associated with expression levels in *SLC22A18* (CMC samples), *MED17* (CMC samples), *NAP1L4* (CMC samples), *SCARNA9* (CMC samples and xQTLServer samples) and *TAF1D* (CMC samples) in the DLPFC (Supplementary Table S5 and Supplementary Fig. S16).

Top SNPs for cyclothymic temperament also took part in chromatin interactions in the adult cortex: *RNU1-91P*, *ZNF195*:*TSSC2*, *SRP14P2*, *SMCO4* in Giusti-Rodriguez et al.^29^ and in neural progenitor cells in GSE87112 database with *ERBB4*, *AC012491.1*, *AIMP2*, *SLC36A4: C14orf2; Lnc-DEUP1-1* (Supplementary Table S6).

#### Functional effects of top SNPs identified for the depressive temperament phenotype

In case of the depressive temperament phenotype, the identified top SNPs did not affect expression levels of genes in the brain (Supplementary Table S7) and did not take part in chromatin interactions with known genes (Supplementary Table S7).

#### Functional effects of top SNPs identified for the irritable temperament phenotype

Top SNPs associated with the irritable temperament did not influence expression levels of any genes according to FUMA (Supplementary Table S9); however, they took part in chromatin interactions in the adult and fetal cortex with *NBEA*^29^, and in the fetal cortex with *RFC3*^29^ (Supplementary Table S10).

#### Functional effects of top SNPs identified for the hyperthymic temperament phenotype

FUMA revealed that top SNPs for hyperthymic temperament influenced expression levels in *HCG27* in DLPFC (in CMC) samples in chromosome 6, and in *SLC4A4* in DLPFC, *HLA-C* in DLPFC (in CMC samples) and *C4A* in DLPFC (in CMC samples) in chromosome 6 (Supplementary Table S11 and Supplementary Fig. S22). Top SNPs for the hyperthymic temperament were also involved in chromatin interactions in neural progenitor cells with *EIF3S3:UTP23* in GSE87112 database (Supplementary Table S12).

## Discussion

Our present study is the first to analyse the genome-wide association of affective temperaments in a general European population sample with precise in-depth phenotyping for these endophenotypes. As affective temperaments can be considered sub-affective manifestations of, and when present in a dominant form, high-risk states for the development of different types of affective illnesses^12^, they may provide a complex approach to understand the genetic background of different affective disorders and mood syndromes yielding clinical relevance for our present results. Although several suggestively significant SNPs and genes with potential relevance for affective disorders and other psychiatric and psychological phenotypes emerged, we most notably identified one SNP in case of the anxious temperament, which survived correction for multiple testing in *ADGRB3* where several other suggestively significant SNPs also underline the potential importance of this gene.

Previously, only one study investigated the genetic background of affective temperaments in a whole-genome analysis approach, in 1263 bipolar patients in the Bipolar Genome Study^14^ identifying 3 genome-wide significant SNPs for the hyperthymic temperament near *MDMI* on chromosome 12 and *FBLNI* on chromosome 22, and 2 significant SNPs on chromosome 1 within neighbouring *INTS7* and *DTL* genes, which led the authors to conclude that temperamental aspects may define clinically and genetically homogeneous bipolar disorder subtypes. Although that study differed from our present one in that they applied a bipolar patient sample as opposed to our general sample, none of the above SNPs or regions have been significant or suggestively significant in our present analysis.

In the present study, we analysed genome-wide associations for each of the five affective temperaments separately and given the significant moderate intercorrelations between them, all models were corrected for the effect of all other temperaments at SNP-based levels. In the next step, as assigning SNPs to genes purely based on their proximity yields only a limited insight into the complexity of the genetic background and little information on the whole spectrum of possible functional effects, and as the majority of identified SNPs are intronic or located in noncoding RNA introns and thus likely influence function of protein- or RNA-coding genes by mechanisms other than amino acid replacement, we carried out functional analysis using FUMA focusing on chromatin interactions and effects on gene expression in the brain. Here we discuss findings related to genes expressed in the brain or implicated in relevant psychiatric or psychological phenotypes in previous analyses.

### Genetic underpinnings of the anxious affective temperament

The most robust finding of the study was identification of a novel intronic SNP in the *ADGRB3* gene on chromosome 6, which survived correction for multiple testing in addition to 21 other intronic variants with suggestive *p*-values within this gene supporting its involvement in anxious temperament. The *ADGRB3* gene is expressed almost exclusively in the brain and encodes the Adhesion G protein-coupled receptor B3, or brain-specific angiogenesis inhibitor 3 (BAI3), a protein with a pivotal role in the central nervous system development, maintenance and plasticity, including regulating synaptic development, axon guidance, synapse formation, myelination, with high expression in the postsynaptic density, especially in the cerebellum, and crucial roles in Purkinje cell synaptogenesis, both during development and adulthood^30–32^. Specifically, the encoded protein controls synaptic connectivity of excitatory inputs, and several SNPs and copy number variations within this gene have previously been associated with different psychiatric disorders including bipolar disorder^33^, schizophrenia^34^ and addiction^35^, as well as with traits such as impulsivity/negative urgency/behavioural disinhibition^36^ and educational attainment^37^. *ADGRB3* shows high expression in the cerebellum and in hippocampal neurons where it acts as a regulator of synapse density and where its knockdown leads to significant impairment in dendrite morphogenesis in mouse models, whereas in a human study the clinical spectrum in patients with biallelic intragenic duplication in *ADGRB3* also included, among other symptoms, anxiety and mood instability, which are associated with hippocampal dysfunction^32,38,39^ and which is specifically in line with our findings that variation in this gene shows association of anxious affective temperament. Further research, focusing on the manipulation of this gene in knockout rodent paradigms, as well as human studies focusing on its association with other anxiety-related endophenotypes and neuropsychiatric symptoms and illnesses should further validate the potential role of this gene and its variation in association with anxiety-related traits and disorders, and eventually the receptor encoded by this gene should be investigated as a potential treatment target.

In gene-based analyses for the anxious temperament, several genes reached suggestive significance after correction for multiple testing, some of which play a role in the central nervous system processes and thus may have potential relevance. Genes with a suggestive significance for the anxious temperament include *FYN*, encoding the proto-oncogene tyrosine protein kinase fyn, which, among several other functions, plays a role in neuronal development and axon guidance, and is implicated in various neural functions and processes. Fyn kinase has, in previous studies, been implicated to play a role in the background of anger/aggression^40^ and in the pathophysiology of major depression^41,42^, bipolar disorder^43^, schizophrenia^44,45^ and Parkinson’s disease^46,47^, and has been suggested as a potential drug target in Alzheimer’s disease^48^. *PIP* encoding prolactin-induced protein has also showed suggestive significance in association with anxious temperament. An SNP in the *PIP* gene in a previous GWAS has been found to be associated with unipolar depression (rs28672333)^49^. *HECTD1*, another gene suggestively associated with the anxious temperament in our present analysis, regulates the expression of *SNAIL*^50^, which in turn regulates neural crest differentiation and neurogenesis^51^. Finally, *ROGDI* also emerged as a suggestive gene in association with anxious temperament. ROGDI encodes a typical leucine zipper protein with an unknown function, with highest expression in the adult brain^52^ and lower expression in the fetal brain and other tissues.

Furthermore, functional prediction analyses implicated top SNPs for the anxious temperament to be associated with the expression of various genes in the central nervous system in areas relevant both for emotional reactivity and affective illnesses, such as *SLC35F2* in the DLPFC, hypothalamus and nucleus accumbens, and implicated in GWASs for unipolar depression, bipolar disorder, schizophrenia, attention deficit hyperactivity disorder (ADHD) and autism spectrum disorder^53^. Top SNPs for the anxious temperament also influence expression of *ACAT1* encoding the acetyl-CoA Acetyltransferase1, the major isoform in the brain, which has been implicated in several neurodegenerative disorders and has recently emerged as a promising target in the treatment of Alzheimer’s disease^54^ and glioblastoma^55^. Top SNPs for the anxious temperament also regulate the expression of *ELMOD1*, which has previously been associated with personality^56^ and delayed discounting measures^57^.

### Genetic underpinnings of the cyclothymic affective temperament

In SNP-based tests in case of the cyclothymic temperament, suggestive SNPs were located either on chromosome 2 within the *ERBB4* gene or on chromosome 11, with 1 out of 6 of these latter SNPs in the *NAP1L4* or *SNORA* genes. One more suggestive SNP was located in the *CSMD1* gene.

*ERBB4* is a member of the Tyr kinase family and encodes a neuregulin surface receptor regulating development of the central nervous system including gene transcription, cell proliferation, differentiation, migration and apoptosis, expressed in interneurons in the frontal cortex and involved in postsynaptic modulation of GABAergic function^58^. Variation in *ERBB4* has been reported in association with serotonin metabolite levels^59^, the openness personality trait of the five-factor model^60^, neuroticism^61–63^, positive affect/wellbeing^63^, intelligence^64,65^, educational attainment^62^, cognitive function^37^, loneliness/isolation/social interaction^66–68^ and depressed affect/unipolar depression/mood disorder^61^. Functional analysis indicated that top SNPs also take part in chromatin interactions with *ERBB4* in the brain further underlying its association with cyclothymic temperament.

*CSMD1*, another gene with a suggestive SNP in the SNP-based tests for cyclothymic temperament, is expressed in the developing central nervous system and shows particular enrichment in the nerve growth cone, and has been suggested as a key regulator of complement activation and inflammation in the developing central nervous system^69^. Variation in *CSMD1* has been suggested in GWASs in association with several potentially relevant phenotypes including chronotype/morningness^62,70–72^, neuroticism^61^, loneliness^73^, impulsivity/negative urgency/behavioural disinhibition^36^, illegal drug consumption^74^, cannabis dependence^75^, alcohol consumption^76^, nicotine dependence^77,78^, cognitive performance^79^, reaction time/cognitive performance^65^, self-reported educational attainment^37^, a combined analysis for five major psychiatric disorders including attention deficit/hyperactivity disorder (ADHD)/major depressive disorder/schizophrenia/autism spectrum disorder (ASD)/bipolar disorder^53^, aggressiveness in ADHD^80^, post-traumatic stress disorder (PTSD)^81^, schizophrenia/schizoaffective disorder/bipolar disorder^82^, psychosis-proneness^83^, paliperidone-efficacy^84^, schizophrenia^44,45,85–92^, suicide attempt in bipolar disorder^93^, eating disorder^94^ and in a common analysis of eight psychiatric disorders (anorexia nervosa, obsessive-compulsive disorder, ADHD, Tourette syndrome, unipolar depression, schizophrenia, autism spectrum disorder and bipolar disorder)^94^.

There were several suggestive hits for cyclothymic temperament at the gene level as well, including *NAP1L4* where a functional effect was also observed and which has been mentioned at a suggestive significance level in association with major depression in a transcriptome-wide analysis^95^. Top SNPs for cyclothymic temperament influenced brain expression of a few potentially relevant genes and participated in chromatin interactions, most notably with *ERBB4* and *SLC36A4*, which latter encodes a sodium-independent electroneutral transporter for tryptophan, proline and alanine, and is also associated with chronotype^71^.

### Genetic underpinnings of the depressive affective temperament

At the SNP level, several suggestively significant SNPs for the depressive temperament were identified in the *SGCZ* gene, which is expressed in several brain regions including highest expression in the cerebellum, basal ganglia and cortex, and has been implicated in the sarcoglycan complex and muscular dystrophy. *SGCZ* has, in previous studies, been associated with some potentially relevant phenotypes including anxiety^61,62^, neuroticism^61,62,96^, cognitive function^65^, mathematical ability^37^, intelligence^64,97^, self-reported educational attainment^37^, depressive symptom and response to antidepressant^98^, schizophrenia^99^ and response to paliperidone^84^.

At the gene level, *UGT2B7* encoding UDP-Glucuronosyltransferase-2B7, a phase II metabolic isoenzyme, also found in the brain showed a suggestive-level association with depressive temperament. For top SNPs in association with depressive temperament, we could not identify relevant effects on gene expression in the brain or chromatin interactions.

### Genetic underpinnings of the irritable temperament

At the SNP level, suggestive SNPs for the irritable temperament were identified on chromosome 17 in the *SHISA6* gene, which encodes a protein involved in the maintenance of high-frequency synaptic transmission in hippocampal CA3-CA1 synapses as an auxiliary α-amino-3-hydroxy-5-methyl-4-isoxazolepropionic acid (AMPA) receptor (AMPAR) subunit, and regulates postsynaptic AMPAR glutamate receptor immobilization keeping the channel in an activated state in the presence of glutamate, preventing synaptic depression and desensitization^100^. Variation in the *SHISA6* gene in GWASs has been implicated in risk-taking behaviour/adventurousness^101^, insomnia^70^, sleep duration^102^ and schizophrenia^88^. In gene-level analyses, suggestive hits included *ENTPD6*, which has been found to be associated with smoking status/smoking initiation^103^, and brain volume of the left pallidum^104^. In functional analyses, no effect of our top SNPs for irritable temperament emerged for influencing gene expression, but chromatin interactions were found with *NBEA* and *RFC3* in the adult and fetal cortices. *NBEA* encodes neurobeachin, lack of which protein in the BEACH domain in neurons was associated with a sharp reduction in synaptic responses as a consequence of reduced surface GABA-A and glutamate receptors, accumulation of immature AMPA receptors, and NMDA, kainite and GABA-A receptors not reaching the synapse, suggesting that Nbea plays a role in regulating basal neurotransmission and targeting of neurotransmitter receptors to synapses^105^. *NBEA* variations in GWASs have been reported in antipsychotic response in schizophrenia^106^, nicotine dependence^78^, self-reported educational attainment^37^, PTSD^76^, and cognitive function^37^.

### Genetic underpinnings of the hyperthymic temperament

At the SNP level, suggestive hits for the hyperthymic temperament were identified on chromosome 4, 8 and 20, and on chromosome 6 in the region of *CDSN* and *PSORSC1* genes. *PSORSC1* is expressed in the brain and has been associated in previous GWASs with neuroticism^63^, risk-taking behaviour^101^, wellbeing/positive affect and wellbeing/life satisfaction, and subjective wellbeing^63,107^, depressive symptom measurement^63^, schizophrenia^44,88,108^, a broad depression or schizophrenia phenotype^109^ and autism spectrum disorder^108^. On the gene level, suggestively significant hits for hyperthymic temperament included *SNX6* and *CANT1* with high expression in the brain, but no relevant phenotypic associations in previous studies. Top SNPs associated with hyperthymic temperament participated in chromatin interactions with a few brain-expressed genes but without previous association with relevant phenotypes.

### Potential transdiagnostic and pathoplastic nature of affective temperaments

Our results indicate that the five distinct affective temperaments have non-overlapping genetic backgrounds; however, several suggestively significant hits were observable for genes which have previously been implicated in association with relevant psychological traits related to the development of mood disorders such as neuroticism, impulsiveness, aggression or wellbeing, as well as in other psychiatric disorders. These two findings, namely distinct genetic underpinnings of the five distinct temperaments and the same disorders these distinct genes confer a risk for, suggest a potentially transdiagnostic endophenotypic nature of the TEMPS affective temperaments. If considering psychiatric disorders other than mood disorders, schizophrenia has been found to have common genetic risk factors with all five temperaments. Addiction may share some genetics with anxious, cyclothymic and irritable temperaments. Autism spectrum disorder has been implicated in relation to risk genes for anxious and hyperthymic temperaments, and PTSD in genes for cyclothymic and irritable temperaments. Distinct temperaments with their distinct genetic underpinnings may represent distinct subgroups within these aetiologically heterogeneous disorders. Stratification of patients or at-risk individuals according to these well-defined endophenotypic pathways, from certain genes through certain affective temperaments to the equifinal endpoint of the disorder, may help in the personalization of preventive and therapeutic strategies for these disorders. However, further longitudinal studies are needed to clarify the mediatory role of affective temperaments between genes and the emergence of these various disorders.

A major weakness of our study is the small sample size highlighted by terming the present analysis a pilot study and which is in part balanced by the careful sample characterization and precise attainment of the investigated phenotype, and multiple analytical approaches focusing on functional analyses besides SNP and gene levels. Furthermore, although precise deep phenotyping of affective temperaments is a strength of the present analysis, affective temperaments are measured based on self-report and are ascertained cross-sectionally, and thus may be influenced by state-like factors such as mood or possible mood disorder episode.

In conclusion, our GWAS focusing on affective temperaments, besides several suggestively significant hits at SNP and gene levels, have most notably identified a new genome-wide significant variant in association with anxious temperament in *AGDRB3* gene. Furthermore, although the five affective temperament phenotypes have non-overlapping genetic backgrounds, we identified several suggestively significant variants previously associated with psychological traits implicated in the development of both mood and other types of psychiatric disorders, implicating the potential transdiagnostic endophenotypic nature of affective temperaments. In the future, our findings may help pave the way for personalized and precision approaches for both prevention and intervention strategies across various psychiatric disorders. Our results should further be tested in larger samples in genome-wide analytical approaches for related phenotypes and candidate gene studies in well-characterized and phenotyped samples, to more precisely establish the role of our identified genes and variants in mood disorders or high-risk states for the development of affective illness, as well as its role as a potential target of therapeutic interventions. Longitudinal studies should also test our findings to understand the potential mediative role of affective temperaments between genes and emergence of various psychiatric disorders.

## Supplementary information

Suppmenetary information

Supplementary File: Quality Control and Imputaton Methods.

Supplementary Figures S1-S23

Supplementary Tables S1-S12.

## Data Availability

Datasets presented in this study are available at 10.6084/m9.figshare.13498536.v1.
