# Supplementary material for: Genetic underpinnings of affective temperaments: a pilot GWAS investigation identifies a new genome-wide significant SNP for anxious temperament in ADGRB3 gene
Source: Transl Psychiatry. 2021 Jun 1;11:337. doi: 10.1038/s41398-021-01436-1 (PMC8169753; doi:10.1038/s41398-021-01436-1)
Supplement: Supplementary file 1 — Suppmenetary information [file 41398_2021_1436_MOESM1_ESM.docx]

**Supplementary file legend**

**Supplementary File 1**. Quality control and imputation methods.

**Supplementary table legends** (uploaded as datasets in .xlsx. format)

**Supplementary Table S1.** Descriptive statistics of the general European sample for genome-wide analysis of affective temperaments

**Supplementary Table S2.** Correlations between affective temperament scales in the present genome-wide analytic study on affective temperaments in a general population sample

**Supplementary Table S3.** Functional effects of top SNPs identified for the anxious temperament phenotype (eQTL)

**Supplementary Table S4.** Functional effects of top SNPs identified for the anxious temperament phenotype (Chromatin interactions)

**Supplementary Table S5.** Functional effects of top SNPs identified for the cyclothymic temperament phenotype (eQTL)

**Supplementary Table S6.** Functional effects of top SNPs identified for the cyclothymic temperament phenotype (Chromatin interactions)

**Supplementary Table S7.** Functional effects of top SNPs identified for the depressive temperament phenotype (eQTL)

**Supplementary Table S8.** Functional effects of top SNPs identified for the depressive temperament phenotype (Chromatin interactions)

**Supplementary Table S9.** Functional effects of top SNPs identified for the irritable temperament phenotype (eQTL)

**Supplementary Table S10.** Functional effects of top SNPs identified for the irritable temperament phenotype (Chromatin interactions)

**Supplementary Table S11.** Functional effects of top SNPs identified for the hyperthymic temperament phenotype (eQTL)

**Supplementary Table S12.** Functional effects of top SNPs identified for the hyperthymic temperament phenotype (Chromatin interactions)

In Tables S3-S12, Yellow highlighting shows central nervous system-relevant findings.

**Supplementary figure legends**

**Supplementary figure S1.** Quantile-quantile plot of genome-wide SNP-based tests for anxious temperament as outcome; with a 95% confidence interval marked (SNP: single-nucleotide polymorphism)

**Supplementary figure S2.** Quantile-quantile plot of genome-wide SNP-based tests for cyclothymic temperament as outcome; with a 95% confidence interval marked. (SNP: single-nucleotide polymorphism)

**Supplementary figure S3.** Quantile-quantile plot of genome-wide SNP-based tests for depressive temperament as outcome; with a 95% confidence interval marked. (SNP: single-nucleotide polymorphism)

**Supplementary figure S4.** Quantile-quantile plot of genome-wide SNP-based tests for irritable temperament as outcome; with a 95% confidence interval marked. (SNP: single-nucleotide polymorphism)

**Supplementary figure S5.** Quantile-quantile plot of genome-wide SNP-based tests for hyperthymic temperament as outcome; with a 95% confidence interval marked. (SNP: single-nucleotide polymorphism)

**Supplementary figure S6.** Quantile-quantile plot of genome-wide gene-based tests for anxious temperament as outcome; with a 95% confidence interval marked.

**Supplementary figure S7.** Quantile-quantile plot of genome-wide gene-based tests for cyclothymic temperament as outcome; with a 95% confidence interval marked.

**Supplementary figure S8.** Quantile-quantile plot of genome-wide gene-based tests for depressive temperament as outcome; with a 95% confidence interval marked.

**Supplementary figure S9.** Quantile-quantile plot of genome-wide gene-based tests for irritable temperament as outcome; with a 95% confidence interval marked.

**Supplementary figure S10.** Quantile-quantile plot of genome-wide gene-based tests for hyperthymic temperament as outcome; with a 95% confidence interval marked.

**Supplementary Figure S11.** Circos plot of gene regulatory role of our top SNPs in all tissues for anxious temperament on chromosome 3.

Inside the zoomed Manhattan plot of SNPs with p<0.05 for anxious temperament and genomic risk loci marked with blue, green color denotes links, mapped genes based on eQTL, and orange color denotes links, mapped genes based on chromatin interaction external databases. Red color denotes genes mapped by both regulatory mechanisms. SNP: single-nucleotide polymorphism; eQTL: expression quantitative trait loci

**Supplementary Figure S12.** Circos plot of gene regulatory role of our top SNPs in all tissues for anxious temperament on chromosome 6.

Inside the zoomed Manhattan plot of SNPs with p<0.05 for anxious temperament and genomic risk loci marked with blue, green color denotes links, mapped genes based on eQTL, and orange color denotes links, mapped genes based on chromatin interaction external databases. Red color denotes genes mapped by both regulatory mechanisms. SNP: single-nucleotide polymorphism; eQTL: expression quantitative trait loci

**Supplementary Figure S13.** Circos plot of gene regulatory role of our top SNPs in all tissues for anxious temperament on chromosome 11.

Inside the zoomed Manhattan plot of SNPs with p<0.05 for anxious temperament and genomic risk loci marked with blue, green color denotes links, mapped genes based on eQTL, and orange color denotes links, mapped genes based on chromatin interaction external databases. Red color denotes genes mapped by both regulatory mechanisms. SNP: single-nucleotide polymorphism; eQTL: expression quantitative trait loci

**Supplementary Figure S14.** Circos plot of gene regulatory role of our top SNPs in all tissues for anxious temperament on chromosome 17.

Inside the zoomed Manhattan plot of SNPs with p<0.05 for anxious temperament and genomic risk loci marked with blue, green color denotes links, mapped genes based on eQTL, and orange color denotes links, mapped genes based on chromatin interaction external databases. Red color denotes genes mapped by both regulatory mechanisms. SNP: single-nucleotide polymorphism; eQTL: expression quantitative trait loci

**Supplementary Figure S15** Circos plot of gene regulatory role of our top SNPs in all tissues for cyclothymic temperament on chromosome 2.

Inside the zoomed Manhattan plot of SNPs with p<0.05 for cyclothymic temperament and genomic risk loci marked with blue, green color denotes links, mapped genes based on eQTL, and orange color denotes links, mapped genes based on chromatin interaction external databases. Red color denotes genes mapped by both regulatory mechanisms. SNP: single-nucleotide polymorphism; eQTL: expression quantitative trait loci

**Supplementary Figure S16** Circos plot of gene regulatory role of our top SNPs in all tissues for cyclothymic temperament on chromosome 11.

Inside the zoomed Manhattan plot of SNPs with p<0.05 for cyclothymic temperament and genomic risk loci marked with blue, green color denotes links, mapped genes based on eQTL, and orange color denotes links, mapped genes based on chromatin interaction external databases. Red color denotes genes mapped by both regulatory mechanisms. SNP: single-nucleotide polymorphism; eQTL: expression quantitative trait loci

**Supplementary Figure S17** Circos plot of gene regulatory role of our top SNPs in all tissues for depressive temperament on chromosome 8.

Inside the zoomed Manhattan plot of SNPs with p<0.05 for depressive temperament and genomic risk loci marked with blue, green color denotes links, mapped genes based on eQTL, and orange color denotes links, mapped genes based on chromatin interaction external databases. Red color denotes genes mapped by both regulatory mechanisms. SNP: single-nucleotide polymorphism; eQTL: expression quantitative trait loci

**Supplementary Figure S18** Circos plot of gene regulatory role of our top SNPs in all tissues for depressive temperament on chromosome 11.

Inside the zoomed Manhattan plot of SNPs with p<0.05 for depressive temperament and genomic risk loci marked with blue, green color denotes links, mapped genes based on eQTL, and orange color denotes links, mapped genes based on chromatin interaction external databases. Red color denotes genes mapped by both regulatory mechanisms. SNP: single-nucleotide polymorphism; eQTL: expression quantitative trait loci

**Supplementary Figure S19** Circos plot of gene regulatory role of our top SNPs in all tissues for irritable temperament on chromosome 13.

Inside the zoomed Manhattan plot of SNPs with p<0.05 for irritable temperament and genomic risk loci marked with blue, green color denotes links, mapped genes based on eQTL, and orange color denotes links, mapped genes based on chromatin interaction external databases. Red color denotes genes mapped by both regulatory mechanisms. SNP: single-nucleotide polymorphism; eQTL: expression quantitative trait loci

**Supplementary Figure S20** Circos plot of gene regulatory role of our top SNPs in all tissues for irritable temperament on chromosome 17.

Inside the zoomed Manhattan plot of SNPs with p<0.05 for irritable temperament and genomic risk loci marked with blue, green color denotes links, mapped genes based on eQTL, and orange color denotes links, mapped genes based on chromatin interaction external databases. Red color denotes genes mapped by both regulatory mechanisms. SNP: single-nucleotide polymorphism; eQTL: expression quantitative trait loci

**Supplementary Figure S21** Circos plot of gene regulatory role of our top SNPs in all tissues for hyperthymic temperament on chromosome 4.

Inside the zoomed Manhattan plot of SNPs with p<0.05 for hyperthymic temperament and genomic risk loci marked with blue, green color denotes links, mapped genes based on eQTL, and orange color denotes links, mapped genes based on chromatin interaction external databases. Red color denotes genes mapped by both regulatory mechanisms. SNP: single-nucleotide polymorphism; eQTL: expression quantitative trait loci

**Supplementary Figure S22** Circos plot of gene regulatory role of our top SNPs in all tissues for hyperthymic temperament on chromosome 6.

Inside the zoomed Manhattan plot of SNPs with p<0.05 for hyperthymic temperament and genomic risk loci marked with blue, green color denotes links, mapped genes based on eQTL, and orange color denotes links, mapped genes based on chromatin interaction external databases. Red color denotes genes mapped by both regulatory mechanisms. SNP: single-nucleotide polymorphism; eQTL: expression quantitative trait loci

**Supplementary Figure S23** Circos plot of gene regulatory role of our top SNPs in all tissues for hyperthymic temperament on chromosome 8.

Inside the zoomed Manhattan plot of SNPs with p<0.05 for hyperthymic temperament and genomic risk loci marked with blue, green color denotes links, mapped genes based on eQTL, and orange color denotes links, mapped genes based on chromatin interaction external databases. Red color denotes genes mapped by both regulatory mechanisms. SNP: single-nucleotide polymorphism; eQTL: expression quantitative trait loci
