# Supplementary material for: Genetic underpinnings of affective temperaments: a pilot GWAS investigation identifies a new genome-wide significant SNP for anxious temperament in ADGRB3 gene
Source: Transl Psychiatry. 2021 Jun 1;11:337. doi: 10.1038/s41398-021-01436-1 (PMC8169753; doi:10.1038/s41398-021-01436-1)
Supplement: Supplementary file 2 — Supplementary File: Quality Control and Imputaton Methods. [file 41398_2021_1436_MOESM2_ESM.docx]

**Methods**

***Supplementary File 1.* - Quality control and imputation methods**

The set of variants were imputed and filtered according to multiple quality control (QC) steps.

Prior to imputation, single-nucleotide biallelic variants of autosomal chromosomes were extracted from the dataset, and their strand alignments were checked against that of the reference data provided by IMPUTE2 (see <https://mathgen.stats.ox.ac.uk/impute/1000GP_Phase3.html>). Imputation was performed using a two-phase process: first, haplotype information was determined using SHAPEIT (<https://mathgen.stats.ox.ac.uk/genetics_software/shapeit/shapeit.html>), then missing variants were imputed from the reference data using IMPUTE2 (<http://mathgen.stats.ox.ac.uk/impute/impute_v2.html>), yielding a total of 2 550 710 variants.

The subsequent QC consisted of the following steps. First, multiallelic and not single-nucleotide variants were excluded, and second, we excluded variants with an imputation score “info” less than 0.5 or “certainty” less than 0.7. After that, filtering of variants and participants was performed. Thresholds for these QC steps were the followings: a minor allele frequency (MAF) minimum of 0.01; iteratively 0.1, 0.05, and 0.01 missingness; a p-value ≥ 1x10^-5^ for the Hardy-Weinberg equilibrium test; an R2 of 0.2 for the linkage disequilibrium (LD) pruning; and an identical-by-descent π^ ≤ 0.1875. We further excluded individuals with a problematic inferred gender, or those who were outliers according to their heterozygosity.
