# Supplementary material for: Genetic underpinnings of affective temperaments: a pilot GWAS investigation identifies a new genome-wide significant SNP for anxious temperament in ADGRB3 gene
Source: Transl Psychiatry. 2021 Jun 1;11:337. doi: 10.1038/s41398-021-01436-1 (PMC8169753; doi:10.1038/s41398-021-01436-1)
Supplement: Supplementary file 3 — Supplementary Figures S1-S23 [file 41398_2021_1436_MOESM3_ESM.docx]

**Results**

**SNP-based results for anxious temperament**


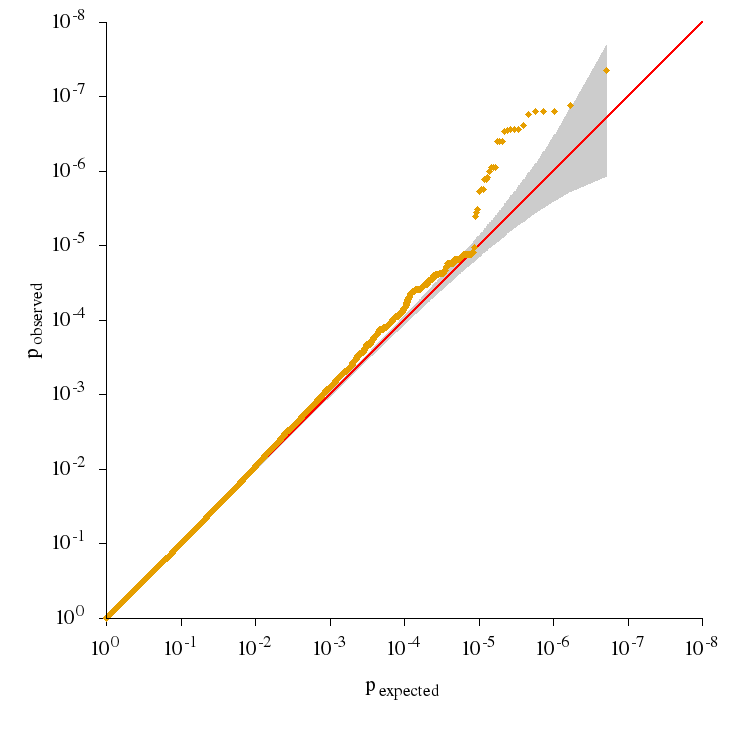


***Supplementary figure S1.* - Quantile-quantile plot of genome-wide SNP-based tests for anxious temperament as outcome; with a 95% confidence interval marked** (SNP: single-nucleotide polymorphism)

**SNP-based results for cyclothymic temperament**


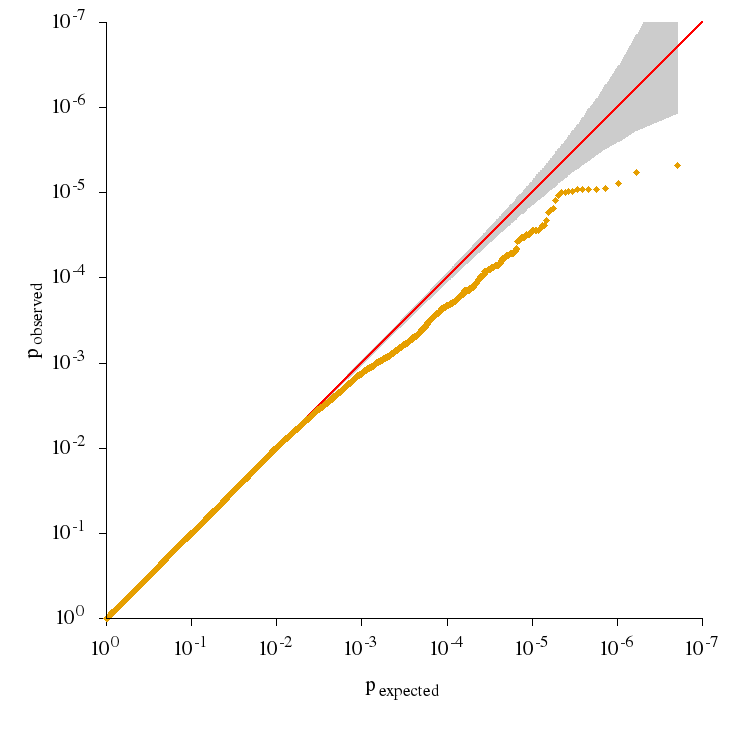


***Supplementary figure S2.* -** **Quantile-quantile plot of genome-wide SNP-based tests for cyclothymic temperament as outcome; with a 95% confidence interval marked.** (SNP: single-nucleotide polymorphism)

**SNP-based results for depressive temperament**


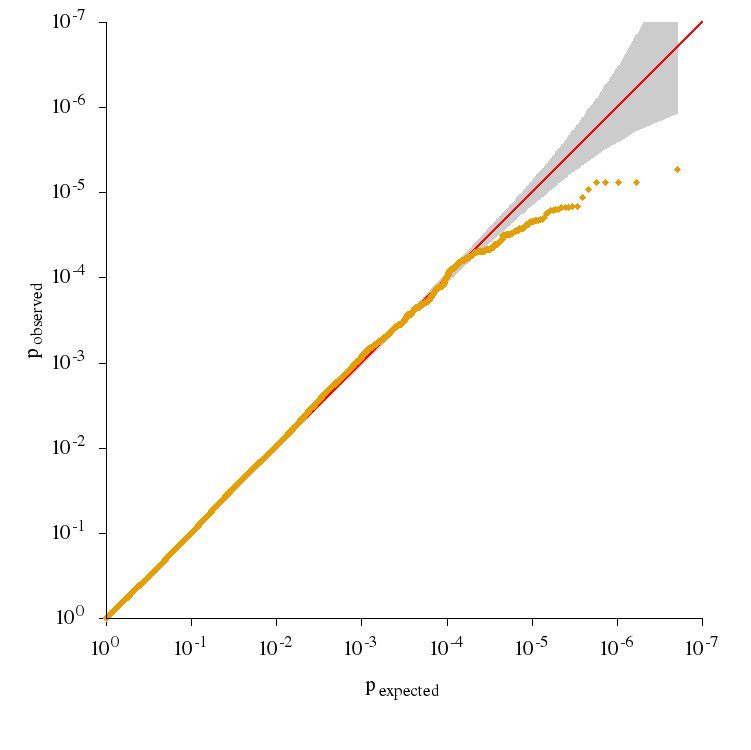


***Supplementary figure S3.*** - **Quantile-quantile plot of genome-wide SNP-based tests for depressive temperament as outcome; with a 95% confidence interval marked.** (SNP: single-nucleotide polymorphism)

**SNP-based results for irritable temperament**


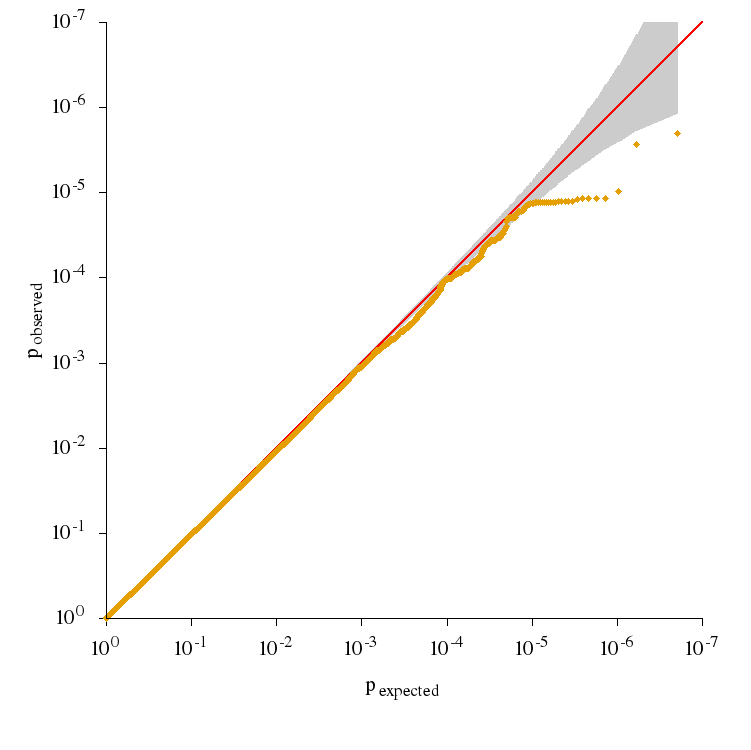


***Supplementary figure S4.* - Quantile-quantile plot of genome-wide SNP-based tests for irritable temperament as outcome; with a 95% confidence interval marked.** (SNP: single-nucleotide polymorphism)

**SNP-based results for hyperthymic temperament**


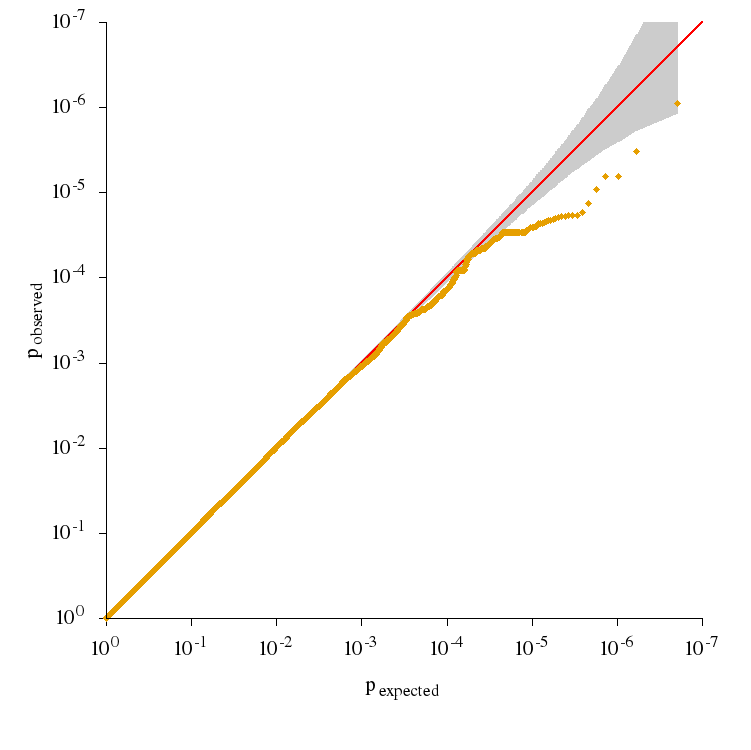


***Supplementary figure S5.* -** **Quantile-quantile plot of genome-wide SNP-based tests for hyperthymic temperament as outcome; with a 95% confidence interval marked.** (SNP: single-nucleotide polymorphism)

**Gene-based based results for anxious temperament**


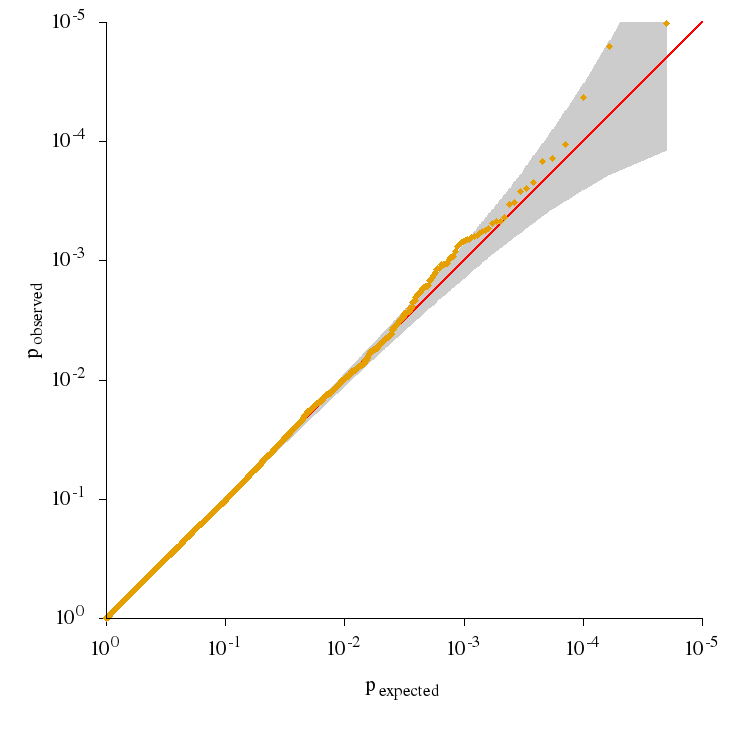


***Supplementary figure S6.* -** **Quantile-quantile plot of genome-wide gene-based tests for anxious temperament as outcome; with a 95% confidence interval marked.**

**Gene-based based results for cyclothymic temperament**


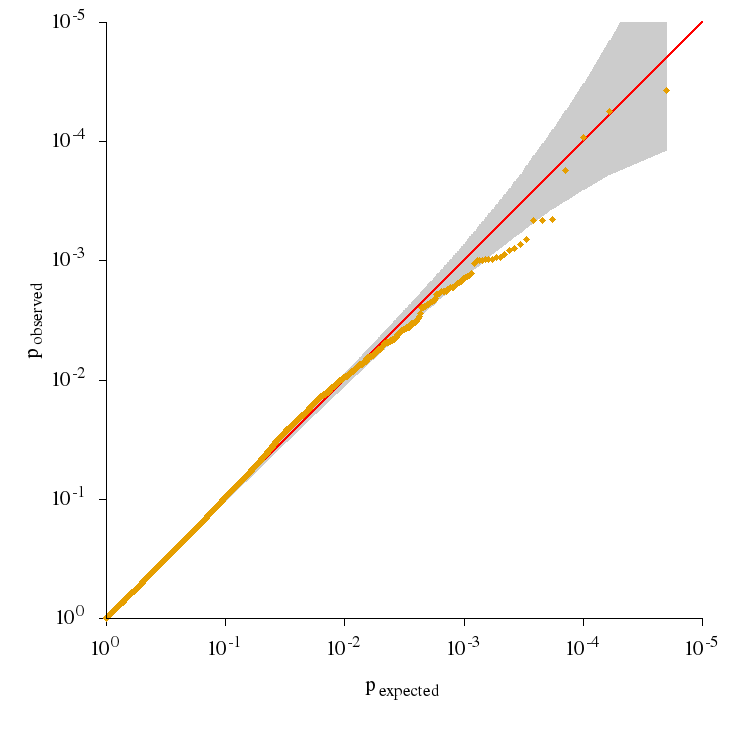


***Supplementary figure S7.* - Quantile-quantile plot of genome-wide gene-based tests for cyclothymic temperament as outcome; with a 95% confidence interval marked.**

**Gene-based based results for depressive temperament**


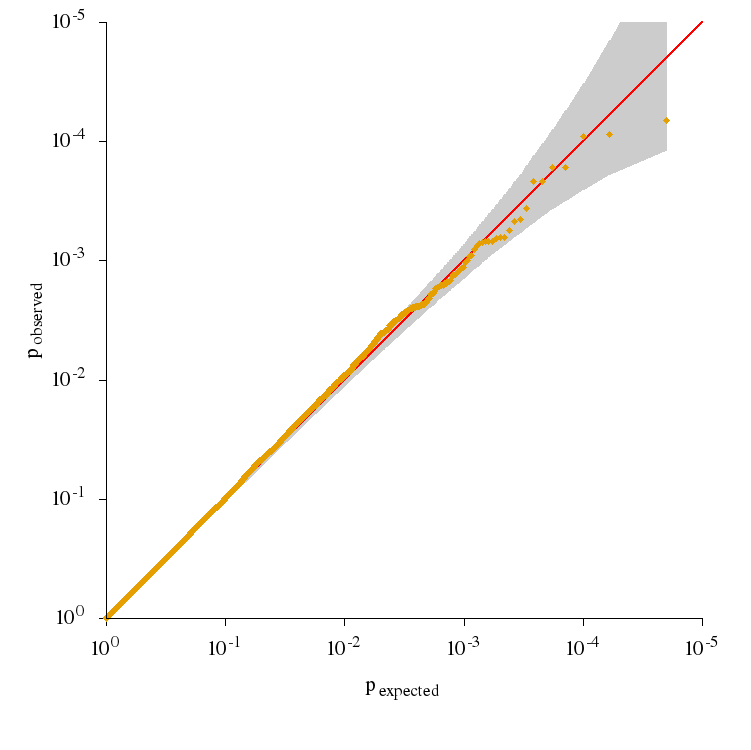


***Supplementary figure S8.*** **- Quantile-quantile plot of genome-wide gene-based tests for depressive temperament as outcome; with a 95% confidence interval marked.**

**Gene-based based results for irritable temperament**


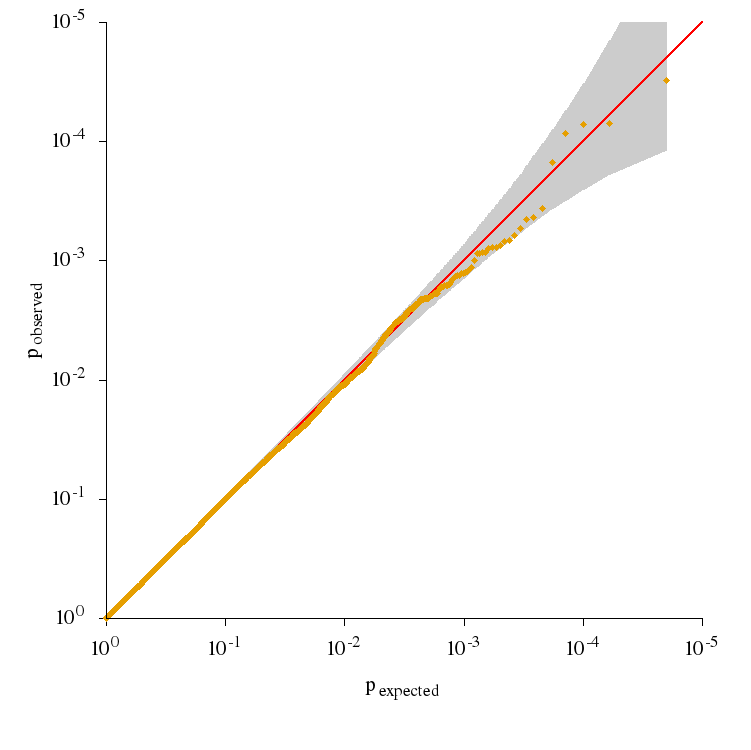


***Supplementary figure S9.* -** **Quantile-quantile plot of genome-wide gene-based tests for irritable temperament as outcome; with a 95% confidence interval marked.**

**Gene-based based results for hyperthymic temperament**


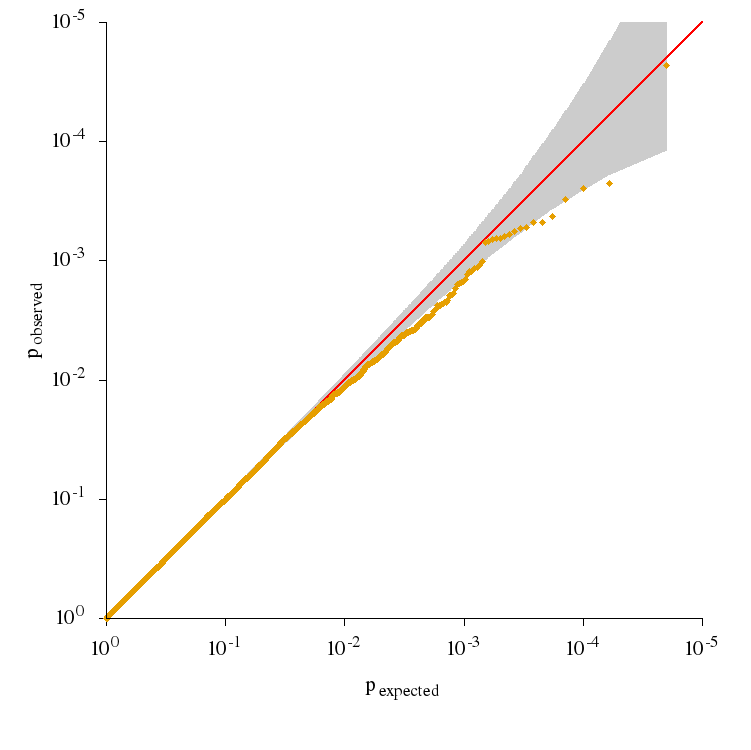


***Supplementary figure S10.*** - **Quantile-quantile plot of genome-wide gene-based tests for hyperthymic temperament as outcome; with a 95% confidence interval marked.**

**Genes regulated in all tissues and cell types by top SNPs of anxious temperament on different chromosomes**


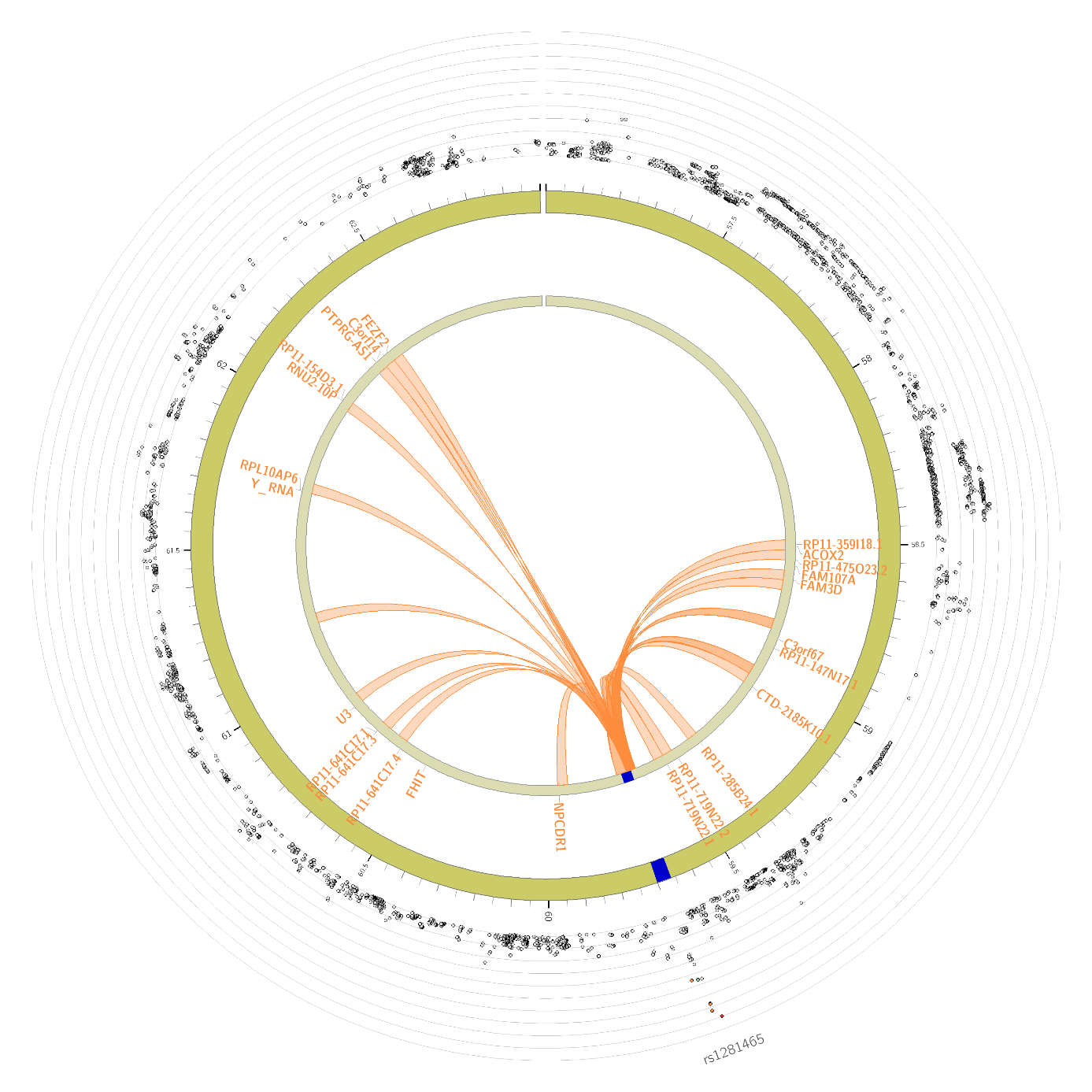


***Supplementary Figure S11* - Circos plot of gene regulatory role of our top SNPs in all tissues for anxious temperament on chromosome 3**. Inside the zoomed Manhattan plot of SNPs with p<0.05 for anxious temperament and genomic risk loci marked with blue, green color denotes links, mapped genes based on eQTL, and orange color denotes links, mapped genes based on chromatin interaction external databases. Red color denotes genes mapped by both regulatory mechanisms. SNP: single-nucleotide polymorphism; eQTL: expression quantitative trait loci


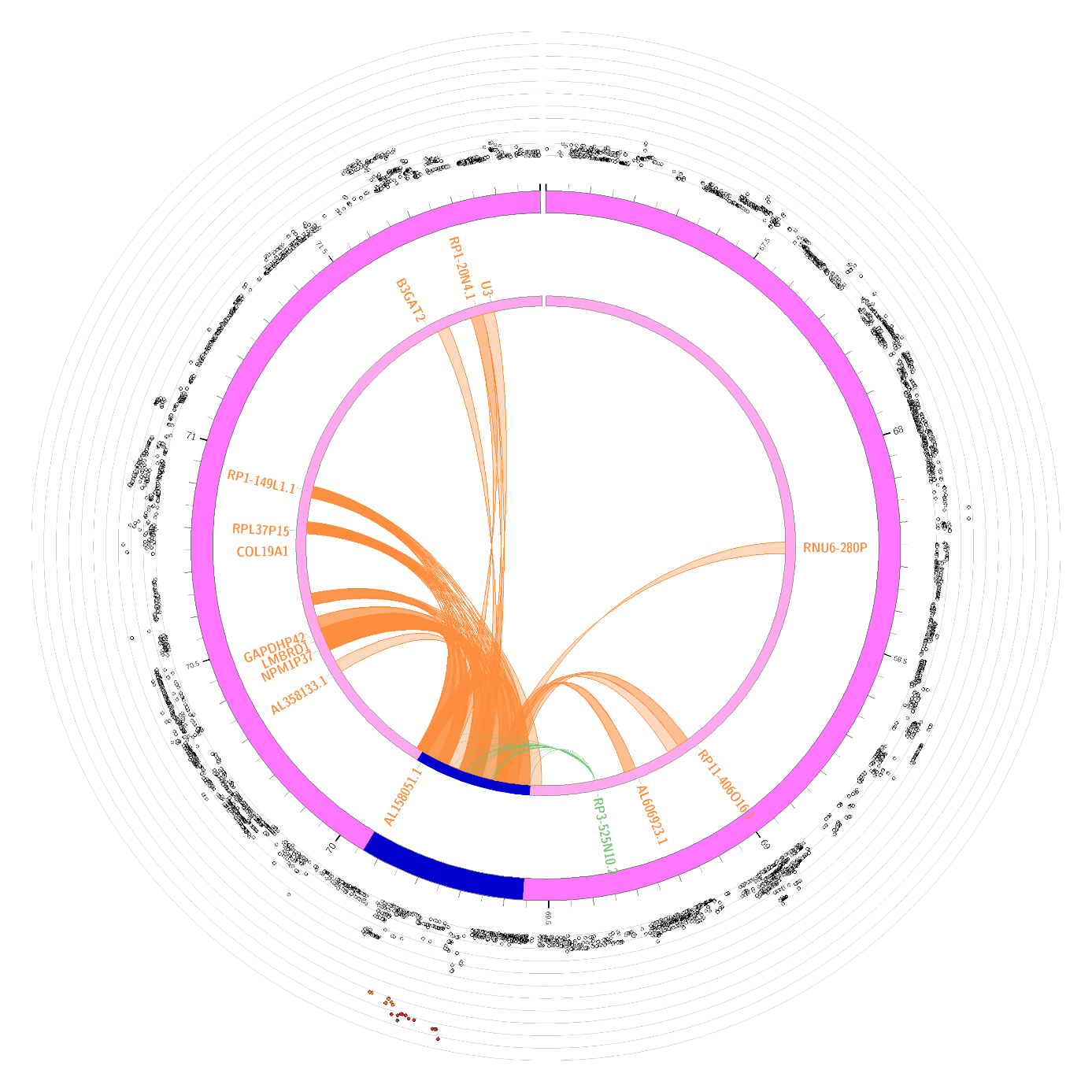


***Supplementary Figure S12* - Circos plot of gene regulatory role of our top SNPs in all tissues for anxious temperament on chromosome 6**. Inside the zoomed Manhattan plot of SNPs with p<0.05 for anxious temperament and genomic risk loci marked with blue, green color denotes links, mapped genes based on eQTL, and orange color denotes links, mapped genes based on chromatin interaction external databases. Red color denotes genes mapped by both regulatory mechanisms. SNP: single-nucleotide polymorphism; eQTL: expression quantitative trait loci


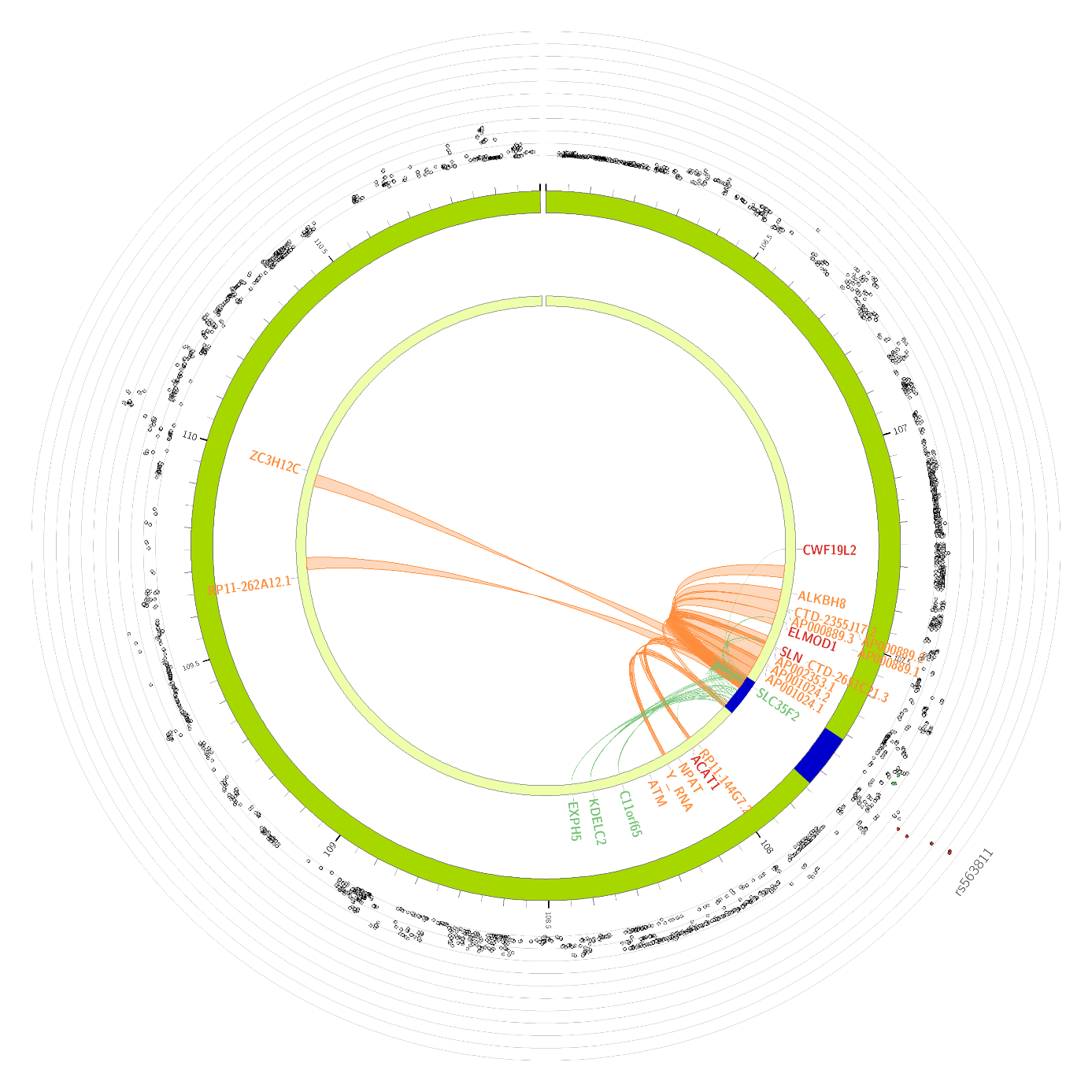


***Supplementary Figure S13* - Circos plot of gene regulatory role of our top SNPs in all tissues for anxious temperament on chromosome 11**. Inside the zoomed Manhattan plot of SNPs with p<0.05 for anxious temperament and genomic risk loci marked with blue, green color denotes links, mapped genes based on eQTL, and orange color denotes links, mapped genes based on chromatin interaction external databases. Red color denotes genes mapped by both regulatory mechanisms. SNP: single-nucleotide polymorphism; eQTL: expression quantitative trait loci


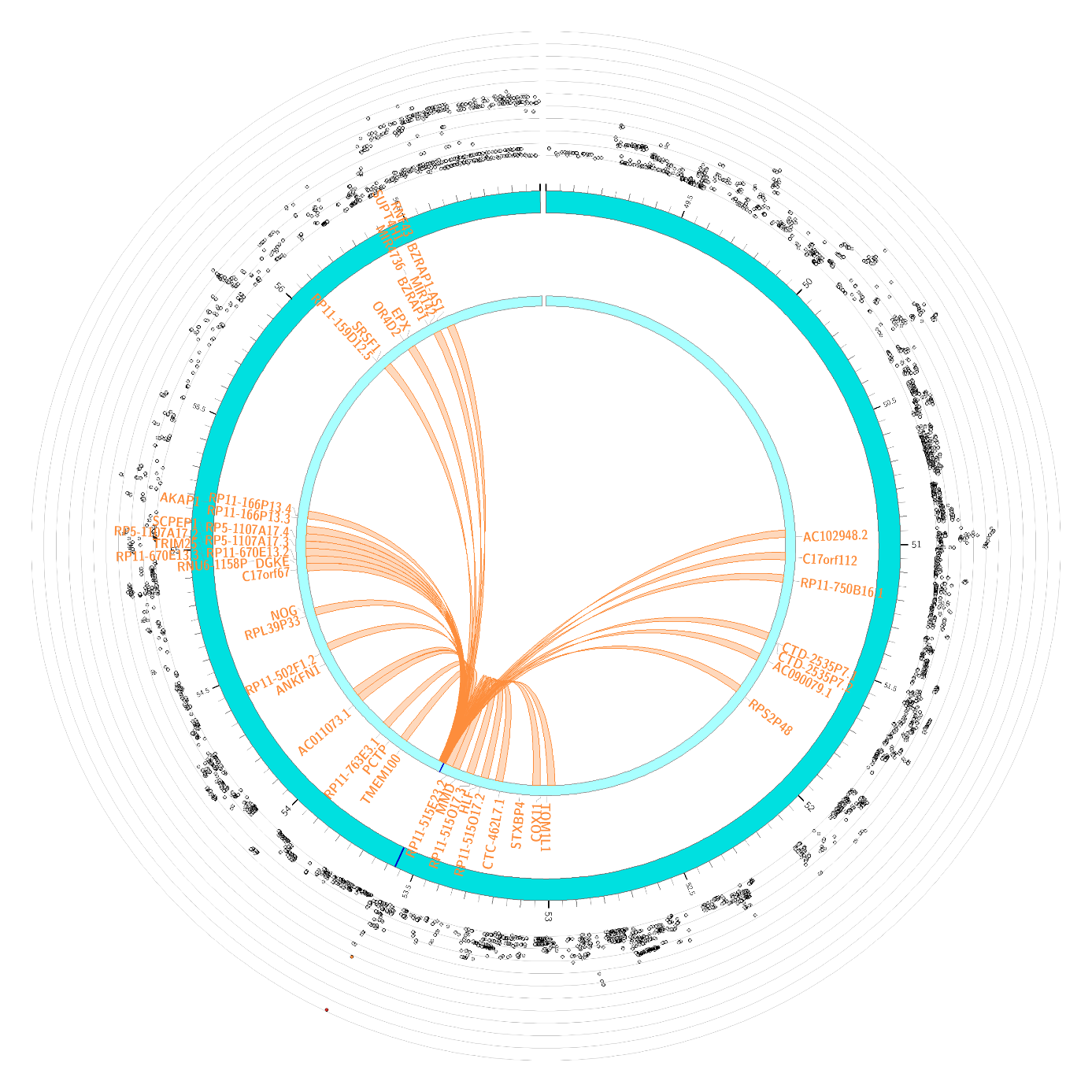


***Supplementary Figure S14* - Circos plot of gene regulatory role of our top SNPs in all tissues for anxious temperament on chromosome 17**. Inside the zoomed Manhattan plot of SNPs with p<0.05 for anxious temperament and genomic risk loci marked with blue, green color denotes links, mapped genes based on eQTL, and orange color denotes links, mapped genes based on chromatin interaction external databases. Red color denotes genes mapped by both regulatory mechanisms. SNP: single-nucleotide polymorphism; eQTL: expression quantitative trait loci

**Genes regulated in all tissues and cell types by top SNPs of cyclothymic temperament on different chromosomes**


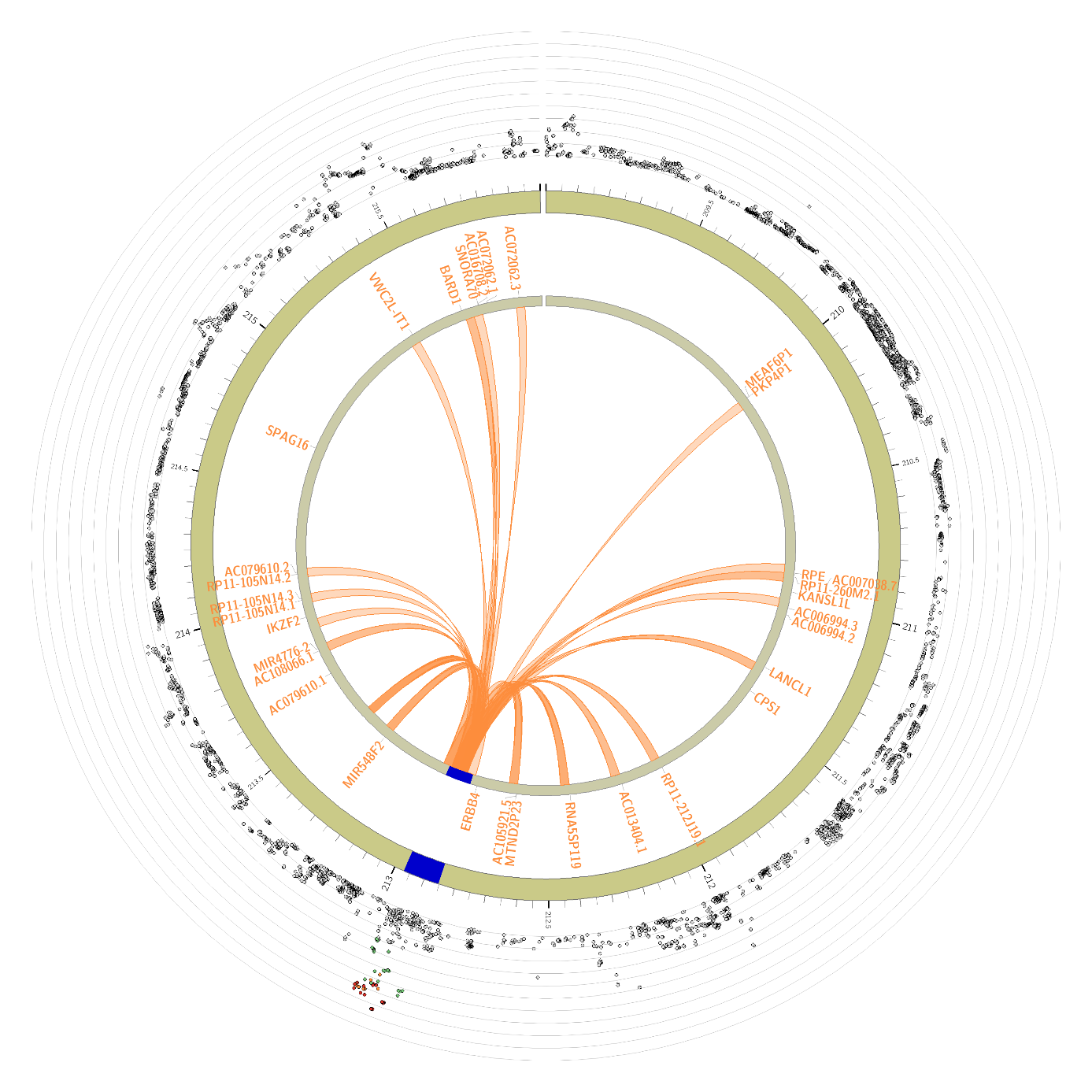


***Supplementary Figure S15* - Circos plot of gene regulatory role of our top SNPs in all tissues for cyclothymic temperament on chromosome 2**. Inside the zoomed Manhattan plot of SNPs with p<0.05 for cyclothymic temperament and genomic risk loci marked with blue, green color denotes links, mapped genes based on eQTL, and orange color denotes links, mapped genes based on chromatin interaction external databases. Red color denotes genes mapped by both regulatory mechanisms. SNP: single-nucleotide polymorphism; eQTL: expression quantitative trait loci


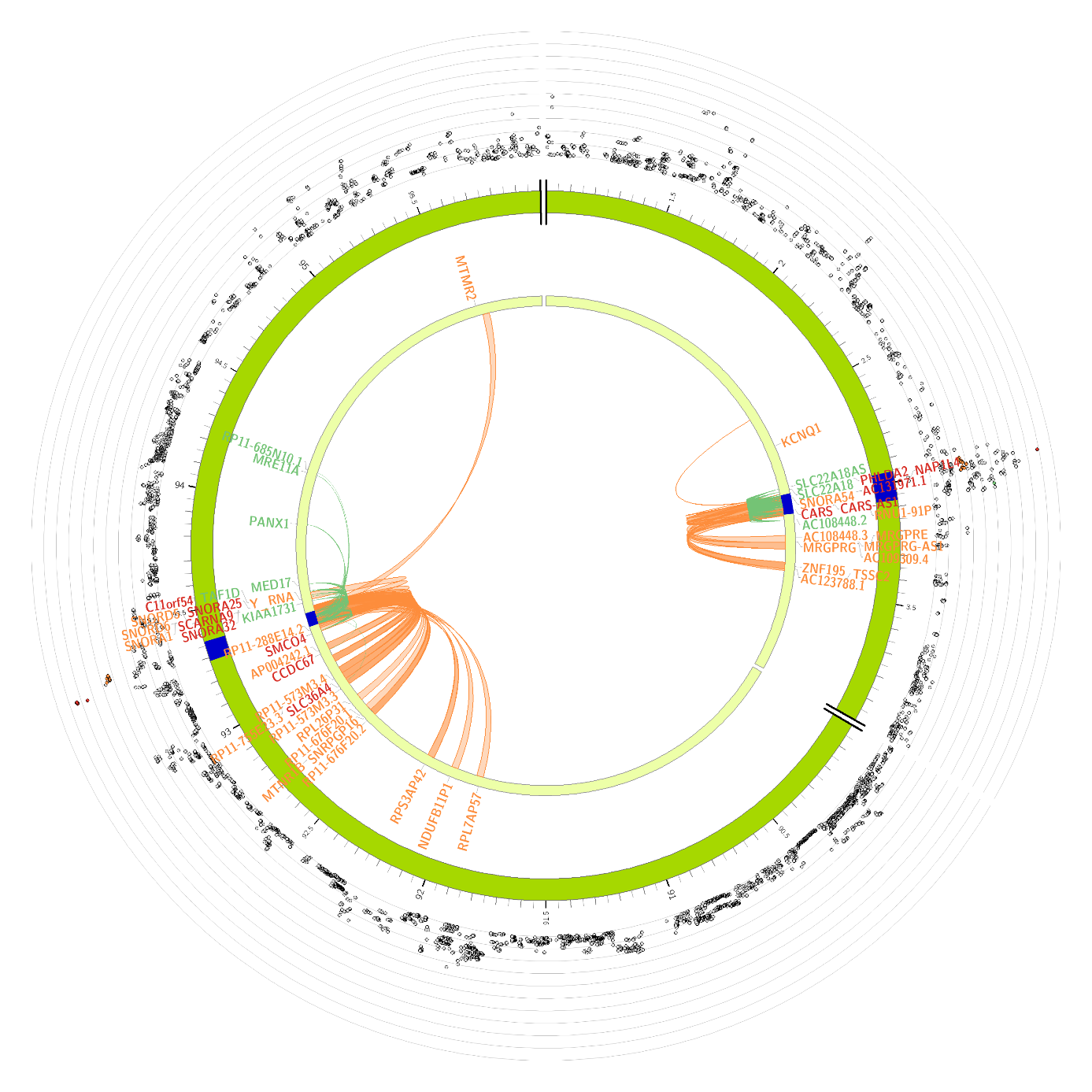


***Supplementary Figure S16* - Circos plot of gene regulatory role of our top SNPs in all tissues for cyclothymic temperament on chromosome 11**. Inside the zoomed Manhattan plot of SNPs with p<0.05 for cyclothymic temperament and genomic risk loci marked with blue, green color denotes links, mapped genes based on eQTL, and orange color denotes links, mapped genes based on chromatin interaction external databases. Red color denotes genes mapped by both regulatory mechanisms. SNP: single-nucleotide polymorphism; eQTL: expression quantitative trait loci

**Genes regulated in all tissues and cell types by top SNPs of depressive temperament on different chromosomes**


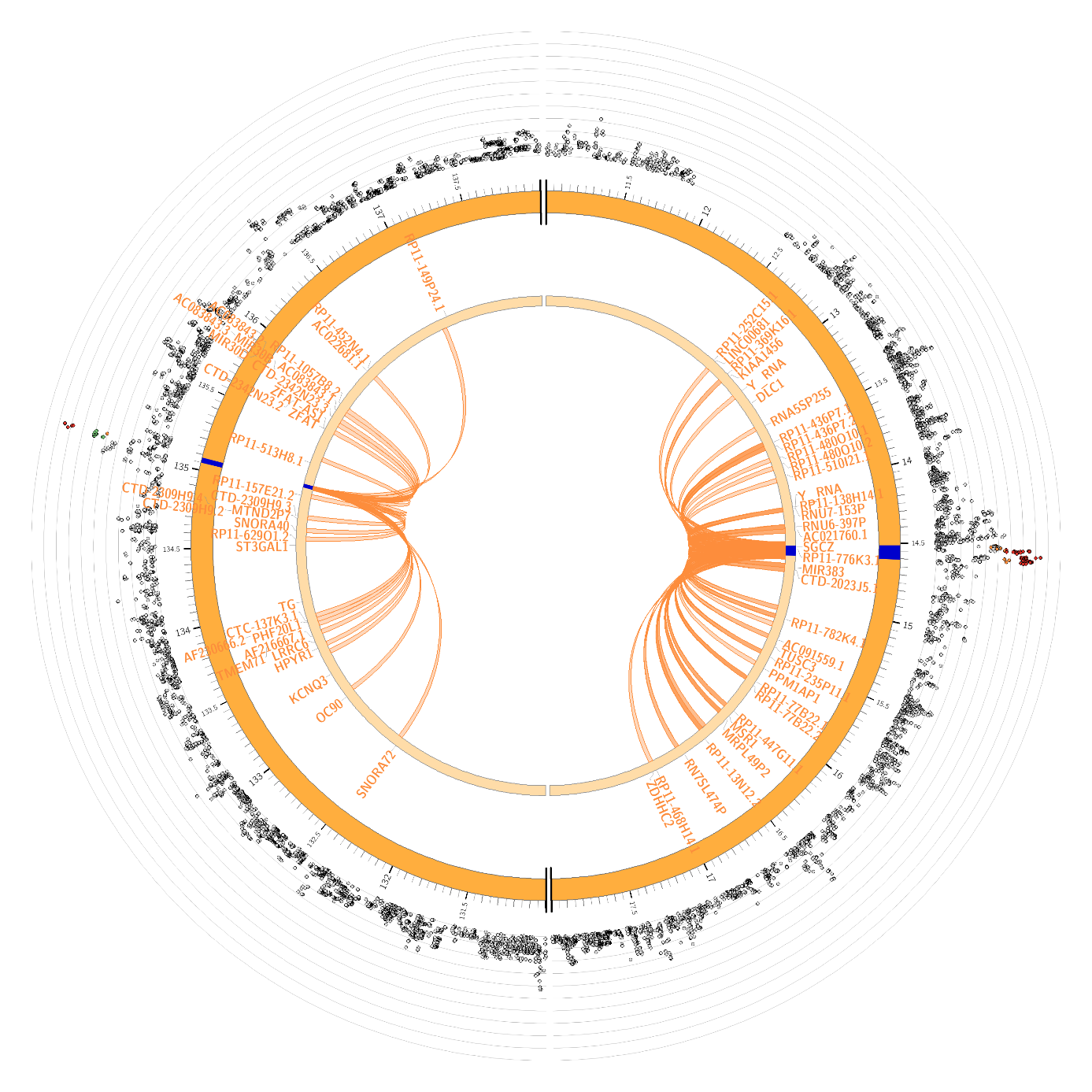


***Supplementary Figure S17* - Circos plot of gene regulatory role of our top SNPs in all tissues for depressive temperament on chromosome 8**. Inside the zoomed Manhattan plot of SNPs with p<0.05 for depressive temperament and genomic risk loci marked with blue, green color denotes links, mapped genes based on eQTL, and orange color denotes links, mapped genes based on chromatin interaction external databases. Red color denotes genes mapped by both regulatory mechanisms. SNP: single-nucleotide polymorphism; eQTL: expression quantitative trait loci


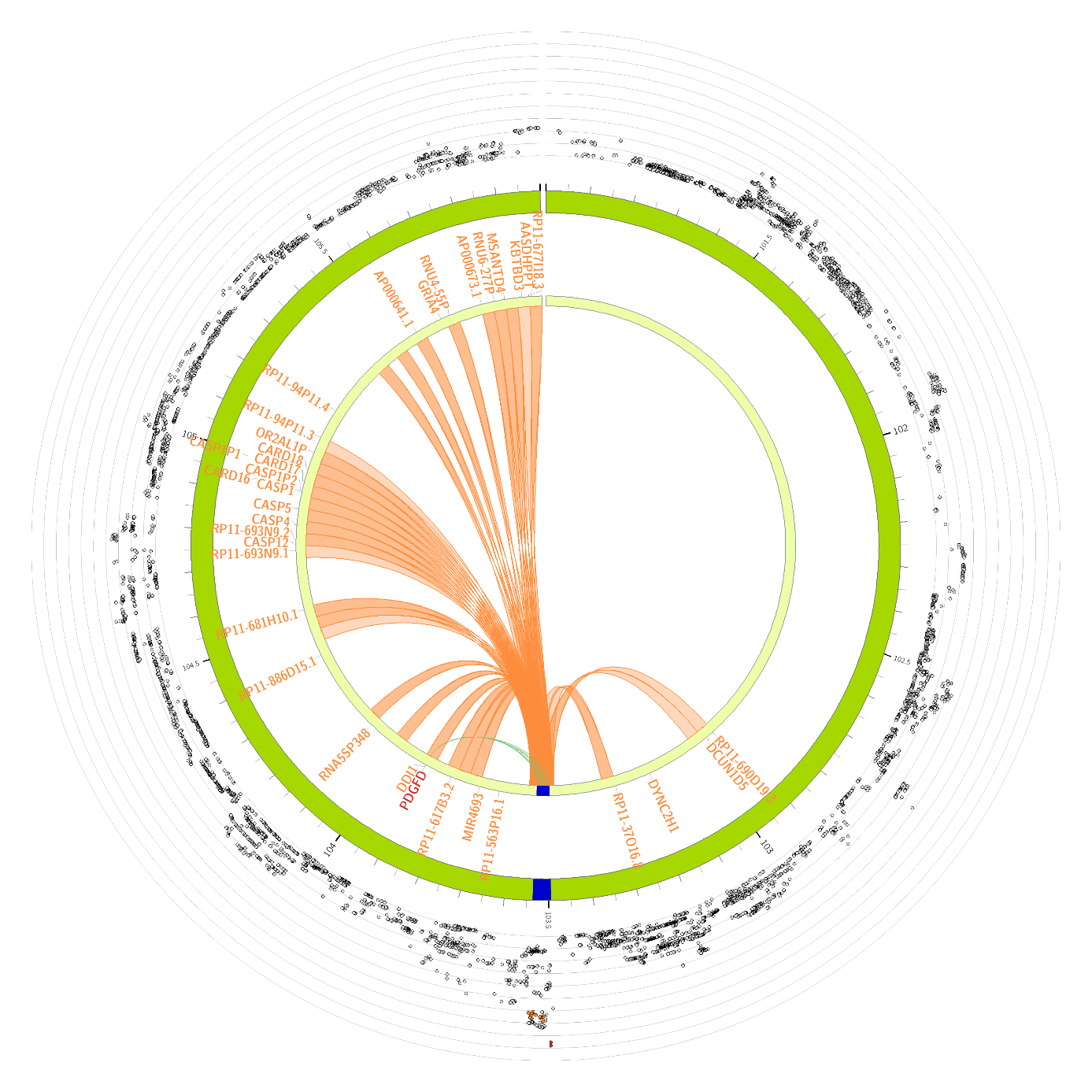


***Supplementary Figure S18* - Circos plot of gene regulatory role of our top SNPs in all tissues for depressive temperament on chromosome 11**. Inside the zoomed Manhattan plot of SNPs with p<0.05 for depressive temperament and genomic risk loci marked with blue, green color denotes links, mapped genes based on eQTL, and orange color denotes links, mapped genes based on chromatin interaction external databases. Red color denotes genes mapped by both regulatory mechanisms. SNP: single-nucleotide polymorphism; eQTL: expression quantitative trait loci

**Genes regulated in all tissues and cell types by top SNPs of irritable temperament on different chromosomes**


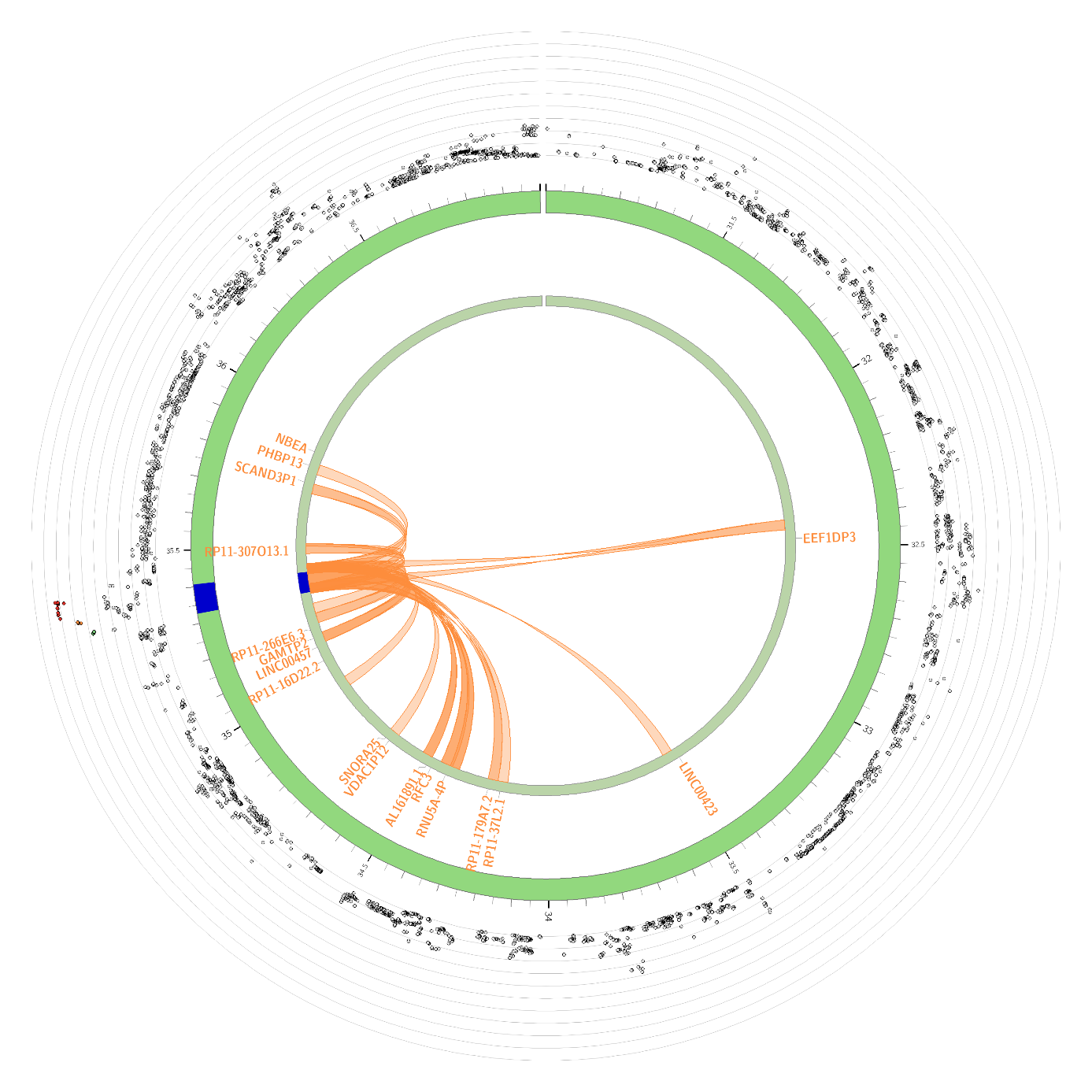


***Supplementary Figure S19* - Circos plot of gene regulatory role of our top SNPs in all tissues for irritable temperament on chromosome 13**. Inside the zoomed Manhattan plot of SNPs with p<0.05 for irritable temperament and genomic risk loci marked with blue, green color denotes links, mapped genes based on eQTL, and orange color denotes links, mapped genes based on chromatin interaction external databases. Red color denotes genes mapped by both regulatory mechanisms. SNP: single-nucleotide polymorphism; eQTL: expression quantitative trait loci


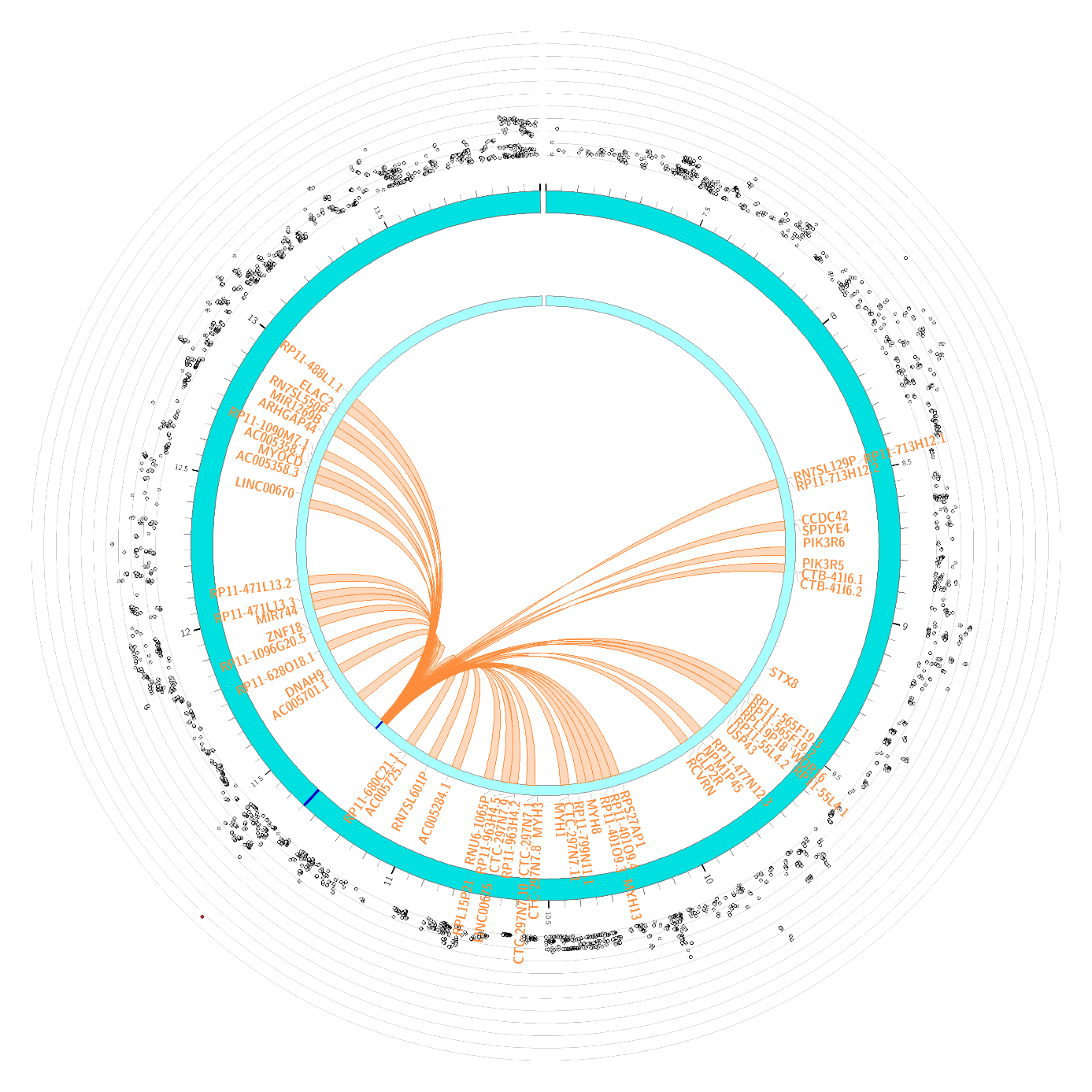


***Supplementary Figure S20* - Circos plot of gene regulatory role of our top SNPs in all tissues for irritable temperament on chromosome 17**. Inside the zoomed Manhattan plot of SNPs with p<0.05 for irritable temperament and genomic risk loci marked with blue, green color denotes links, mapped genes based on eQTL, and orange color denotes links, mapped genes based on chromatin interaction external databases. Red color denotes genes mapped by both regulatory mechanisms. SNP: single-nucleotide polymorphism; eQTL: expression quantitative trait loci

**Genes regulated in all tissues and cell types by top SNPs of hyperthymic temperament on different chromosomes**


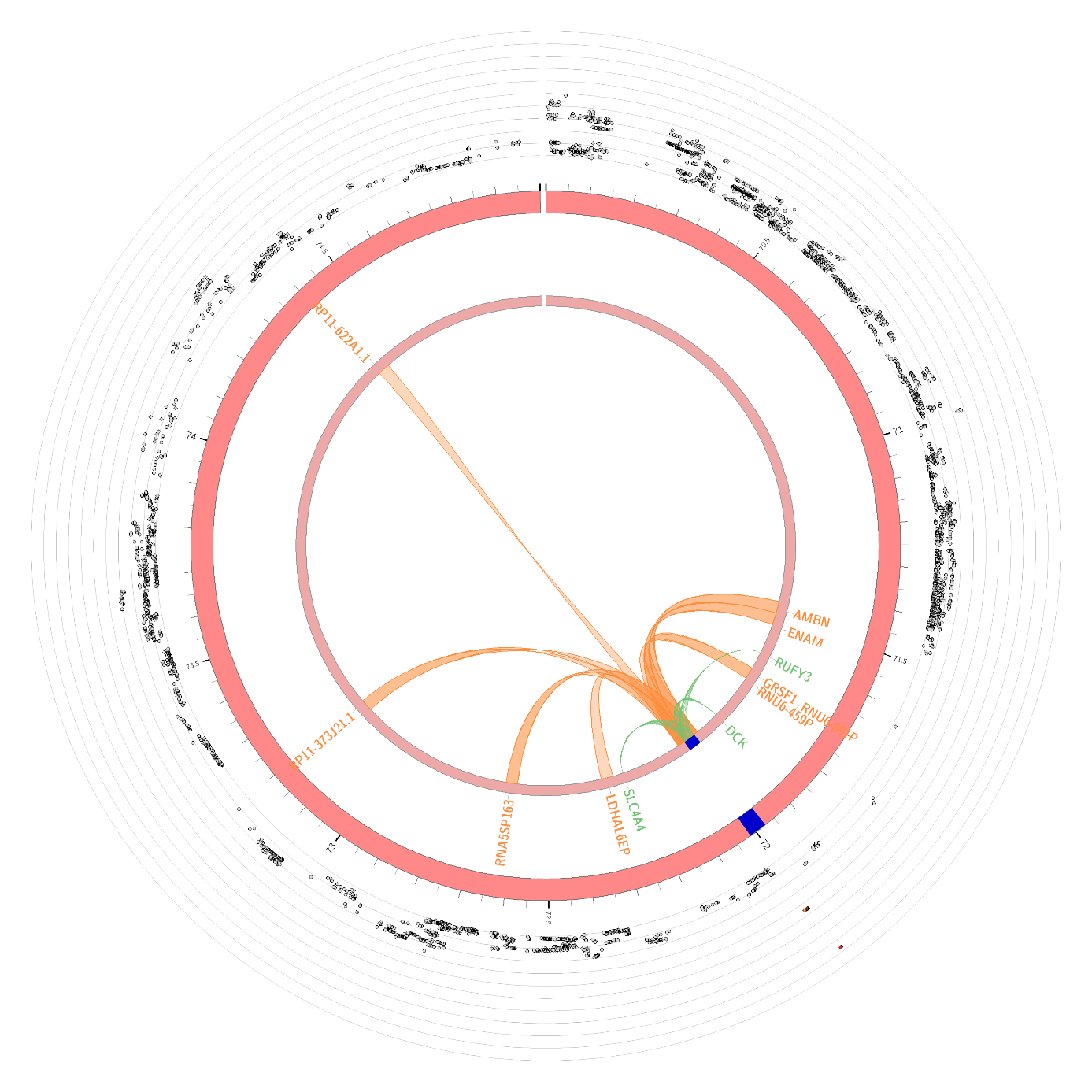


***Supplementary Figure S21* - Circos plot of gene regulatory role of our top SNPs in all tissues for hyperthymic temperament on chromosome 4**. Inside the zoomed Manhattan plot of SNPs with p<0.05 for hyperthymic temperament and genomic risk loci marked with blue, green color denotes links, mapped genes based on eQTL, and orange color denotes links, mapped genes based on chromatin interaction external databases. Red color denotes genes mapped by both regulatory mechanisms. SNP: single-nucleotide polymorphism; eQTL: expression quantitative trait loci


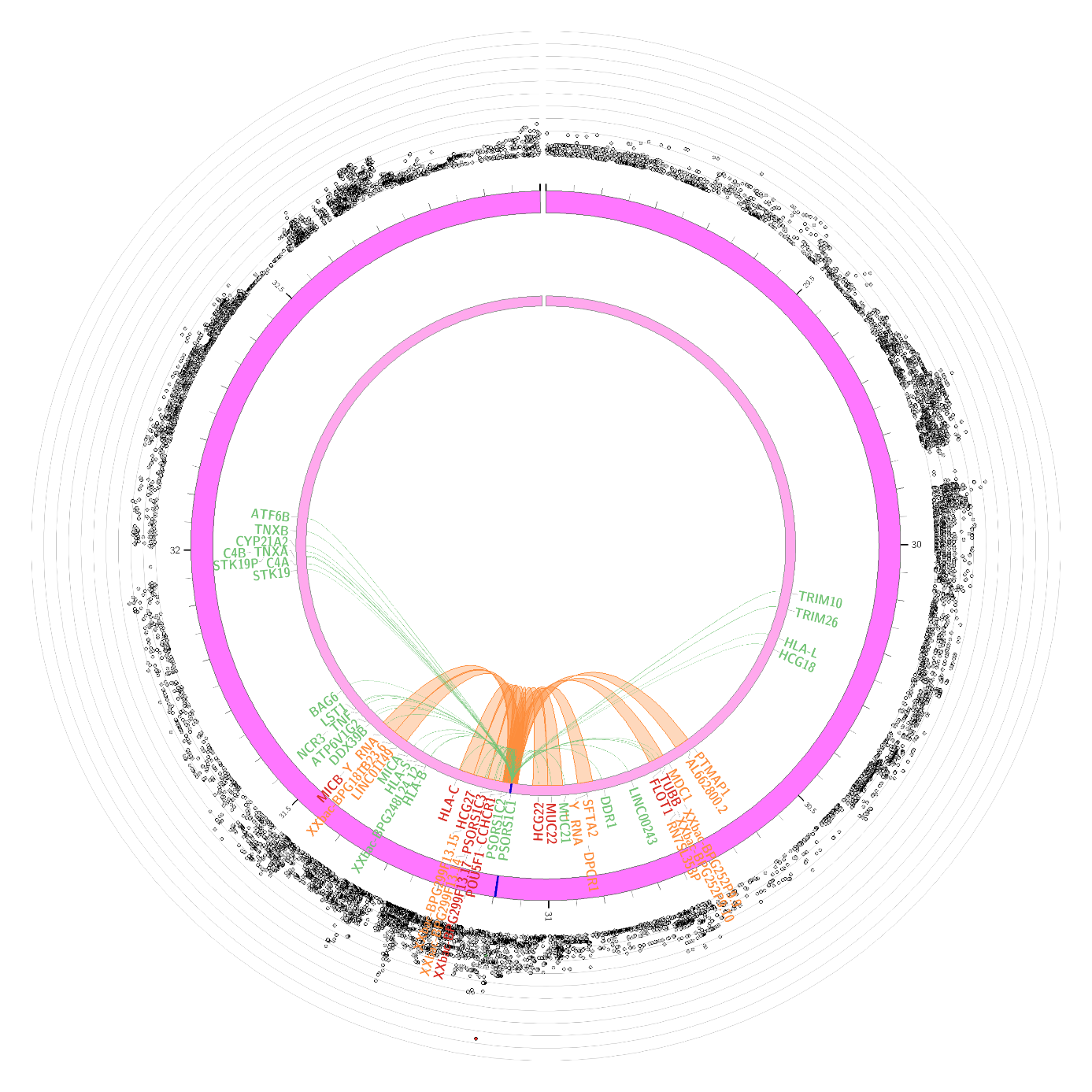


***Supplementary Figure S22* - Circos plot of gene regulatory role of our top SNPs in all tissues for hyperthymic temperament on chromosome 6**. Inside the zoomed Manhattan plot of SNPs with p<0.05 for hyperthymic temperament and genomic risk loci marked with blue, green color denotes links, mapped genes based on eQTL, and orange color denotes links, mapped genes based on chromatin interaction external databases. Red color denotes genes mapped by both regulatory mechanisms. SNP: single-nucleotide polymorphism; eQTL: expression quantitative trait loci


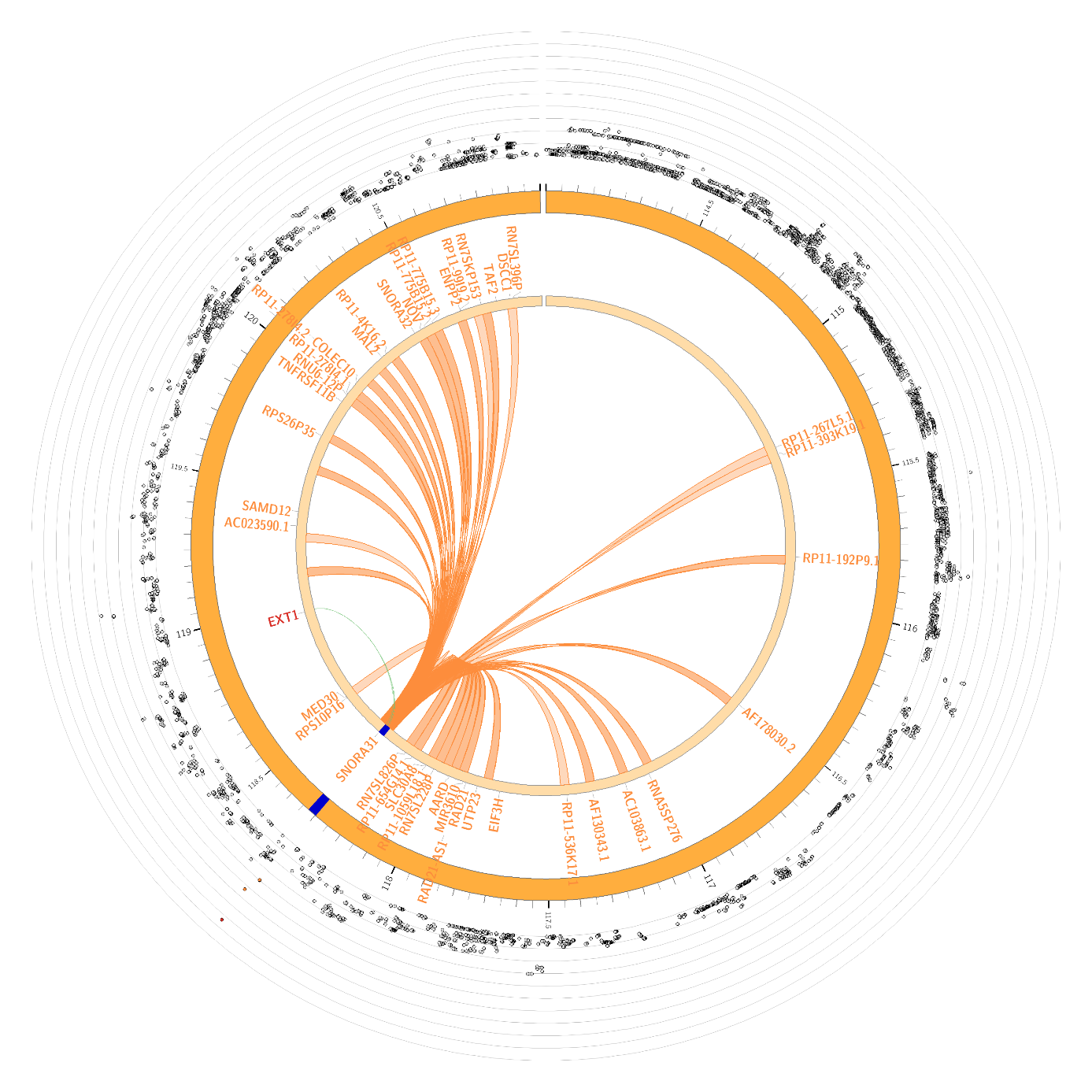


***Supplementary Figure S23* - Circos plot of gene regulatory role of our top SNPs in all tissues for hyperthymic temperament on chromosome 8**. Inside the zoomed Manhattan plot of SNPs with p<0.05 for hyperthymic temperament and genomic risk loci marked with blue, green color denotes links, mapped genes based on eQTL, and orange color denotes links, mapped genes based on chromatin interaction external databases. Red color denotes genes mapped by both regulatory mechanisms. SNP: single-nucleotide polymorphism; eQTL: expression quantitative trait loci
